# New records of ant species from Yunnan, China

**DOI:** 10.3897/zookeys.477.8775

**Published:** 2015-01-26

**Authors:** Cong Liu, Benoit Guénard, Francisco Hita Garcia, Seiki Yamane, Benjamin Blanchard, Da-Rong Yang, Evan Economo

**Affiliations:** 1 Okinawa Institute of Science and Technology Graduate University, Okinawa, 904-0495, Japan; 2 School of Biological Sciences, The University of Hong Kong, Pokfulam Road, Hong Kong; 3 Haruyama-cho 1054-1, Kagoshima-shi, 899-2704, Japan; 4 Committee on Evolutionary Biology, University of Chicago, Chicago, Illinois, USA; 5 Key Laboratory of Tropical Forest Ecology, Xishuangbanna Tropical Botanical Garden, Chinese Academy of Sciences, Kunming, China

**Keywords:** China, Formicidae, new records, Xishuangbanna, Yunnan

## Abstract

As with many other regions of the world, significant collecting, curation, and taxonomic efforts will be needed to complete the inventory of China’s ant fauna. This is especially true for the highly diverse tropical regions in the south of the country, where moist tropical forests harbor high species richness typical of the Southeast Asian region. We inventoried ants in the Xingshuangbanna prefecture, Yunnan, in June 2013, using a variety of methods including Winkler extraction and hand collection to sample ant diversity. We identified 213 species/morphospecies of ants from 10 subfamilies and 61 genera. After identification of 148 valid species of the 213 total species collected, 40 species represent new records for Yunnan province and 17 species are newly recorded for China. This increases the total number of named ant species in Yunnan and China to 447 and 951 respectively. The most common species collected were *Brachyponera luteipes* and *Vollenhovia emeryi*. Only one confirmed exotic species *Strumigenys membranifera*, was collected, although several others were potentially introduced by humans. These results highlight the high biodiversity value of the region, but also underscore how much work remains to fully document the native myrmecofauna.

## Introduction

The understanding of regional and global patterns of insect diversity is limited by our incomplete accounting of Earth’s species, especially for tropical regions where species richness peaks in most taxonomic groups. This is also true for Formicidae, an ecologically dominant insect family comprising at least 15,000 described species (Bolton 2014). Despite the ubiquity and ecological importance of ants (Hölldobler and Wilson 1990), many tropical regions remain undersampled even at the generic level (Guénard and Dunn 2012). Compiling and curating complete and accurate species checklists for all regions of the world should be a priority in biodiversity research, especially for diverse insect groups.

Towards that end, here we present the results of an ant survey conducted during the summer of 2013 in the area of Xishuangbanna, Yunnan Province, in the south of China. In particular, our goal here is to document new records of ant species detected in Yunnan, and some new records for China as a whole. The geographic location of Yunnan (ranging from 21.15°N to 29.20°N of latitude) and its topography (elevation range from < 100m to 6740m) render it the most diverse province of China in terms of ant diversity (406 species) (Guénard and Dunn 2012). The same is true for other taxa, such as plants (Li and Walker 1986, Mutke and Barthlott 2005), tiger beetles (Wu and Shook 2007), butterflies (Xie et al. 2009), or amphibians (Chen and Bi 2007). Xishuangbanna prefecture is located in the tropical southwestern region of Yunnan province, bordering Laos and Myanmar, and has been identified as the most diverse region of Yunnan (Long 1995). The ant fauna of Xishuangbanna has been the subject of three studies (Xu 1998, 1999, 2002) and new species are regularly described from this prefecture (e.g. Guénard et al. 2013, Xu et al. 2014a, b). According to Xu’s survey (2002), the myrmecofauna of Xishuangbanna consists of approximately 262 species, which constitute about 65% of the total number of species recorded for Yunnan province.

While elements of China’s ant fauna may be undocumented due to a lack of sampling in certain geographic regions, there are many taxa likely hidden in areas that have been sampled historically. In particular, methods targeting specifically subterranean or leaf litter ants have been rarely used in China, which as a result might bias our detection of ant species from specific strata. One of the most successful sampling techniques for collecting leaf litter ants, Winkler extraction, which is now commonly used for ant fauna surveys all over the world (Olson 1991, Fisher 1999, Agosti et al. 2000, Martelli et al. 2004, Vasconcelos and Lopes 2008, Ivanov and Keiper 2009), has only been used once in China (Hong Kong in Fellowes 1996) to the best of our knowledge. In this study we used Winkler extraction as a standardized collection technique for the first time in order to survey the leaf litter ant fauna of Xishuangbanna. Based predominantly on this highly successful sampling technique, our diversity survey revealed 40 new species records for Yunnan including 17 new records for China. Here we present those new records, as well as their known global distributions by using data information aggregated by the Global Ant BioInformatics project (GABI, Guénard et al. in prep).

## Material and methods

Ant specimens were collected from primary forest, secondary forest and rubber plantation habitats near Menglun town, Xishuangbanna Prefecture, Yunnan Province, China during a survey in June 2013. Ants from leaf litter of multiple sites were collected and extracted by mini Winkler extractors for 72 hours using the shuffling method as described in Guénard and Lucky (2011). Ants were also collected by hand on the ground, lower vegetation, and tree trunks.

Samples were first sorted to morphospecies in alcohol, and up to three representatives of each morphospecies per sample were point-mounted. Each mounted specimen was assigned a unique specimen code, in this case a CASENT number, and traditional locality and collection labels. All mounted and alcohol-preserved ant specimens are currently located in EPE’s lab at the Okinawa Institute of Science and Technology Graduate University. Extended depth of field specimen images were taken with an incorporated Leica DFC400 digital camera mounted on a Leica M205C stereomicroscope through the Leica Application Suite V4 software. All specimens were identified to genus using Bolton’s key (Bolton 1994), and then identified to species using available keys (see results section) as well as the digital resources on AntWeb (http://www.antweb.org). All the specimen data are freely available on AntWeb.

Species distributions presented in the following maps are based on records reported here at the country level, or for the larger countries (China, India, Japan), at the first administration level. For large islands (e.g. Borneo, Sumatra, New Guinea) that form natural biogeographic units, we used the island boundary instead of political boundaries similar to a previous study (Guénard and Dunn 2012).

## Results

Over 20000 specimens from 61 genera and 213 valid species and morphospecies were collected during this ant diversity survey (Table 1). A total of 40 new species records are presented for Yunnan province. Seventeen of these are recorded for the first time from China. The newly detected species belong to 15 genera from eight subfamilies. It is beyond the scope of the current paper to perform a comprehensive review/revision of the ant fauna of Yunnan Province, which would require much more geographically comprehensive sampling. Here, we present species accounts for the described ant species found during our survey that were previously unknown to Yunnan, supplementing other recently published checklists of the myrmecofauna of the region (Guénard and Dunn 2012).

**Table 1.** Ant species (Formicidae) collected from Xishuangbanna, Yunnan in 2013.

Species	Collection record^1^
**Aenictinae**	
*Aenictus artipus* Wilson, 1964	N*
*Aenictus hodgsoni* Forel, 1901	N
*Aenictus maneerati* Jaitrong & Yamane, 2013	N*
*Aenictus paradentatus* Jaitrong, Yamane & Tasen, 2012	N*
*Aenictus thailandianus* Terayama & Kubota, 1993	N
*Aenictus* clm01	
*Aenictus* clm04	
**Amblyoponinae**	
*Bannapone scrobiceps* Guénard, Blanchard, Liu, Yang & Economo, 2013	N*
*Mystrium camillae* Emery, 1889	
**Cerapachyinae**	
*Cerapachys* clm01	
*Cerapachys sulcinodis* Emery, 1889	
*Cerapachys typhlus* (Roger, 1861)	
**Dolichoderinae**	
*Chronoxenus wroughtonii* (Forel, 1985)	
*Dolichoderus affinis* Emery, 1889	
*Dolichoderus laotius* Santschi, 1920	N*
*Dolichoderus squamanodus* Xu, 2001	
*Dolichoderus thoracicus* (Smith, 1860)	
*Iridomyrmex anceps* (Roger, 1863)	
*Tapinoma indicum* Forel, 1895	
*Tapinoma melanocephalum* (Fabricius, 1793)	
*Tapinoma* clm04	
*Technomyrmex albipes* (Smith, 1861)	
*Technomyrmex horni* Forel, 1912	
*Technomyrmex pratensis* (Smith, 1860)	N
**Ectatomminae**	
*Gnamptogenys costata* (Emery, 1989)	N*
*Gnamptogenys bicolor* (Emery, 1989)	
*Gnamptogenys treta* Lattke, 2004	N*
**Formicinae**	
*Acropyga nipponensis* Terayama, 1985	
*Anoplolepis gracilipes* (Smith, 1857)	
*Camponotus lasiselene* Wang & Wu, 1994	
*Camponotus mitis* (Smith, 1858)	
*Camponotus parius* Emery, 1889	
*Camponotus singularis* Smith, 1858	
*Camponotus* clm02	
*Camponotus* clm03	
*Camponotus* clm04	
*Camponotus* clm07	
*Camponotus* clm08	
*Camponotus* clm09	
*Echinopla cherapunjiensis* Bharti & Gul, 2012	N
*Gesomyrmex kalshoveni* Wheeler, W.M. 1929	N*
*Lepisiota opaca* (Forel, 1892)	
*Lepisiota rothneyi* (Forel, 1894)	
*Myrmoteras binghamii* Forel, 1893	
*Myrmoteras cuneonodum* Xu, 1998	
*Nylanderia* clm01	
*Nylanderia* clm02	
*Nylanderia* clm03	
*Nylanderia* clm04	
*Nylanderia* clm05	
*Nylanderia* clm06	
*Oecophylla smaragdina* (Fabricius, 1775)	
*Paraparatrechina* clm01	
*Paraparatrechina* clm02	
*Paraparatrechina* clm03	
*Paraparatrechina* clm04	
*Plagiolepis* clm01	
*Polyrhachis armata* (Le Guillou, 1842)	
*Polyrhachis bicolor* Mayr, 1862	
*Polyrhachis bihamata* (Drury, 1773)	
*Polyrhachis furcata* Emery, 1889	
*Polyrhachis halidayi* Emery, 1889	
*Polyrhachis hippomanes* Smith, 1861	
*Polyrhachis illaudata* Walker, 1859	
*Polyrhachis illaudata pauperata* Emery, 1889	
*Prenolepis naoroji* Forel, 1902	
*Prenolepis sphingthoraxa* Zhou & Zheng, 1998	N
*Pseudolasius cibdelus* Wu & Wang, 1992	
*Pseudolasius emeryi* Forel, 1915	
*Pseudolasius silvestrii* Wheeler, 1927	
**Myrmicinae**	
*Acanthomyrmex luciolae* Emery, 1893	
*Aphaenogaster beccarii* Emery, 1887	
*Aphaenogaster feae* Emery, 1889	
*Aphaenogaster* clm05	
*Cardiocondyla wroughtonii* (Forel, 1890)	
*Carebara affinis* (Jerdon, 1851)	
*Carebara altinoda* (Xu, 2003)	
*Carebara bruni* (Forel, 1913)	
*Carebara diversa* (Jerdon, 1851)	
*Carebara melasolena* (Zhou & Zheng, 1997)	N
*Carebara* clm01	
*Carebara* clm05	
*Carebara* clm06	
*Carebara* clm07	
*Carebara* clm08	
*Carebara* clm09	
*Carebara* clm10	
*Carebara* clm11	
*Carebara* clm12	
*Carebara* clm13	
*Cataulacus granulatus* (Latreille, 1802)	
*Crematogaster dohrni* Mayr, 1879	
*Crematogaster ferrarii* Emery, 1888	
*Crematogaster millardi* Forel, 1902	
*Crematogaster osakensis* Forel, 1900	
*Crematogaster politula* Forel, 1902	
*Crematogaster rothneyi* Mayr, 1879	
*Crematogaster* clm05	
*Crematogaster* clm09	
*Crematogaster* clm10	
*Crematogaster* clm11	
*Dilobocondyla fouqueti* Santschi, 1910	
*Kartidris ashima* Xu & Zheng, 1995	
*Lophomyrmex quadrispinosus* (Jerdon, 1851)	
*Lordomyrma idianale* Taylor, 2012	
*Meranoplus laeviventris* Emery, 1889	
*Monomorium chinense* Santschi, 1925	
*Monomorium pharaonis* (Linnaeus, 1758)	
*Monomorium* clm01	
*Monomorium* clm02	
*Monomorium* clm05	
*Monomorium* clm06	
*Myrmecina curvispina* Zhou, Huang & Ma L., 2008	N
*Myrmecina guangxiensis* Zhou, 2001	N
*Pheidole hongkongensis* Wheeler, 1928	N
*Pheidole noda* Smith, 1874	
*Pheidole pieli* Santschi, 1925	
*Pheidole plagiaria* Smith, 1860	N
*Pheidole planifrons* Santschi, 1920	N
*Pheidole roberti* Forel, 1902	
*Pheidole rugithorax* Eguchi, 2008	N
*Pheidole sagei* Forel, 1902	
*Pheidole smythiesii* Forel, 1902	N
*Pheidole tumida* Eguchi, 2008	N
*Pheidole vieti* Eguchi, 2008	N*
*Pheidole zoceana* Santschi, 1925	N
*Pheidole* clm03	
*Pheidole* clm04	
*Pheidole* clm07	
*Pheidole* clm12	
*Pheidole* clm13	
*Pheidole* clm16	
*Pheidole* clm18	
*Pheidole* clm22	
*Pheidole* clm13	
*Pristomyrmex brevispinosus* Emery, 1887	
*Pristomyrmex hamatus* Xu & Zhang, 2002	
*Pristomyrmex punctatus* (Smith, 1860)	
*Recurvidris recurvispinosa* (Forel, 1890)	
*Recurvidris kemneri* (Wheeler & Wheeler, 1954)	N*
*Solenopsis jacoti* Wheeler, 1923	
*Strumigenys ailaoshana* (Xu & Zhou, 2004)	
*Strumigenys dyschima* (Bolton, 2000)	N*
*Strumigenys exilirhina* Bolton, 2000	
*Strumigenys feae* Emery, 1895	
*Strumigenys kichijo* (Terayama, Lin & Wu, 1996)	N
*Strumigenys lyroessa* (Roger, 1862)	
*Strumigenys membranifera* Emery, 1869	
*Strumigenys mitis* (Brown, 2000)	N
*Strumigenys mutica* (Brown, 1949)	
*Strumigenys nanzanensis* Lin & Wu, 1996	
*Strumigenys nepalensis* Baroni Urbani & De Andrade, 1994	N*
*Strumigenys rallarhina* Bolton, 2000	N
*Strumigenys sauteri* (Forel, 1912)	N
*Tetramorium aptum* Bolton, 1977	
*Tetramorium ciliatum* Bolton, 1977	
*Tetramorium difficile* Bolton, 1977	N*
*Tetramorium flavipes* Emery, 1893	N*
*Tetramorium kheperra* (Bolton, 1976)	
*Tetramorium kraepelini* Forel, 1905	
*Tetramorium nipponense* Wheeler, 1928	
*Tetramorium parvispinum* (Emery, 1893)	N
*Tetramorium polymorphum* Yamane & Jaitrong, 2011	N*
*Tetramorium tonganum* Mayr, 1870	N
*Tetramorium* clm03	
*Tetramorium* clm10	
*Tetramorium* clm18	
*Tetramorium* clm19	
*Vollenhovia emeryi* Wheeler, 1906	
**Ponerinae**	
*Anochetus graeffei* Mayr, 1870	
*Anochetus mixtus* Radchenko, 1993	
*Anochetus myops* Emery, 1893	
*Anochetus* clm04	
*Brachyponera luteipes* (Mayr, 1862)	
*Diacamma* clm01	
*Ectomomyrmex astutus* (Smith, 1858)	
*Ectomomyrmex leeuwenhoeki* (Forel, 1886)	
*Ectomomyrmex lobocarenus* (Xu, 1995)	
*Ectomomyrmex* clm01	
*Ectomomyrmex* clm02	
*Ectomomyrmex* clm03	
*Ectomomyrmex* clm04	
*Emeryopone melaina* Xu, 1998	
*Hypoponera* clm01	
*Hypoponera* clm02	
*Hypoponera* clm03	
*Hypoponera* clm04	
*Hypoponera* clm05	
*Hypoponera* clm06	
*Hypoponera* clm07	
*Leptogenys birmana* Forel, 1900	
*Leptogenys chinensis* (Mayr, 1870)	
*Leptogenys crassicornis* Emery, 1895	
*Leptogenys diminuta* (Smith, 1857)	
*Leptogenys lucidula* Emery, 1895	
*Leptogenys mengzii* Xu, 2000	
*Leptogenys* clm01	
*Leptogenys* clm02	
*Leptogenys* clm09	
*Myopias hania* Xu & Liu, 2011	
*Odontomachus* clm01	
*Odontoponera denticulata* (Smith, 1858)	N
*Platythyrea parallela* (Smith, 1859)	
*Pseudoneoponera rufipes* (Forel, 1911)	
**Proceratinae**	
*Discothyrea clavicornis* Emery, 1897	N*
*Discothyrea kamiteta* Kubota & Terayama, 1999	N
*Probolomyrmex longiscapus* Xu & Zeng, 2000	
*Proceratium deelemani* Perrault, 1981	N*
**Pseudomyrmecinae**	
*Tetraponera amargina* Xu & Chai, 2004	
*Tetraponera allaborans* (Walker, 1859)	
*Tetraponera attenuata* Smith, 1877	
*Tetraponera concava* Xu & Chai, 2004	

1 N = New to Yunnan province; N* = New to China.

## Species accounts

### Aenictus artipus

Taxon classificationAnimaliaHymenopteraFormicidae

Wilson, 1964

Figure 1

#### Material examined.

CHINA, Yunnan, Xishuangbanna: Man Sai village (21.858°N, 101.277°E), Rubber plantation, 12.vi.2013, 5 workers, 705m, Winkler sifting, B. Guénard, B. Blanchard and C. Liu; Man Sai village (21.860°N, 101.278°E), Secondary forest, 12.vi.2013, 18 workers, 680m, Winkler sifting, B. Guénard, B. Blanchard and C. Liu.

#### Distribution.

**Figure 1.**
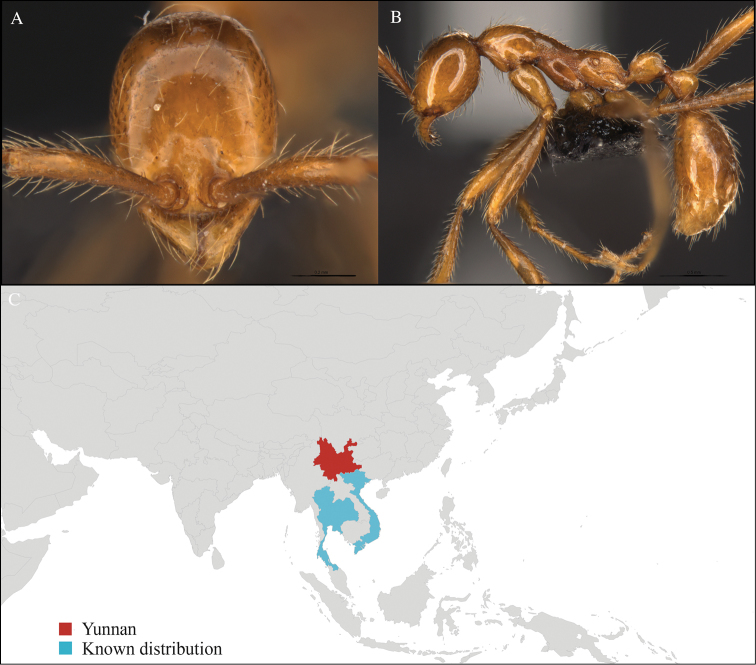
*Aenictus artipus* worker, CASENT0717199. **A** Head in front view **B** Mesosoma in profile view **C** Global distribution map.

Yunnan (new record), Vietnam and Thailand (Figure 1C). This collection represents the northern-most record of *Aenictus artipus*.

#### Taxonomic note.

*Aenictus artipus* belongs to the *Aenictus wroughtonii* species group and can be easily identified with the identification key provided by Jaitrong et al. (2010).

#### Natural history.

*Aenictus artipus* has been collected from leaf litter in various habitats such as secondary forest and rubber plantation located near natural secondary forest. In addition, *Aenictus artipus* has also been found in different habitats such as montane evergreen forest, savanna forest, evergreen forest and disturbed forest (Jaitrong et al. 2010).

### Aenictus hodgsoni

Taxon classificationAnimaliaHymenopteraFormicidae

Forel, 1901

Figure 2

#### Material examined.

CHINA, Yunnan, Xishuangbanna: Xishuangbanna Tropical Botanical Garden (known as ‘XTBG’) (21.919°N, 101.270°E), Secondary forest, 08.vi.2013, 12 workers, 610m, Hand collection, B. Guénard, B. Blanchard and C. Liu; Kilometer 55 station (21.966°N, 101.203°E), Secondary forest, 13.vi.2013, 40 workers, 825m, Winkler sifting, B. Guénard, B. Blanchard and C. Liu.

#### Distribution.

**Figure 2.**
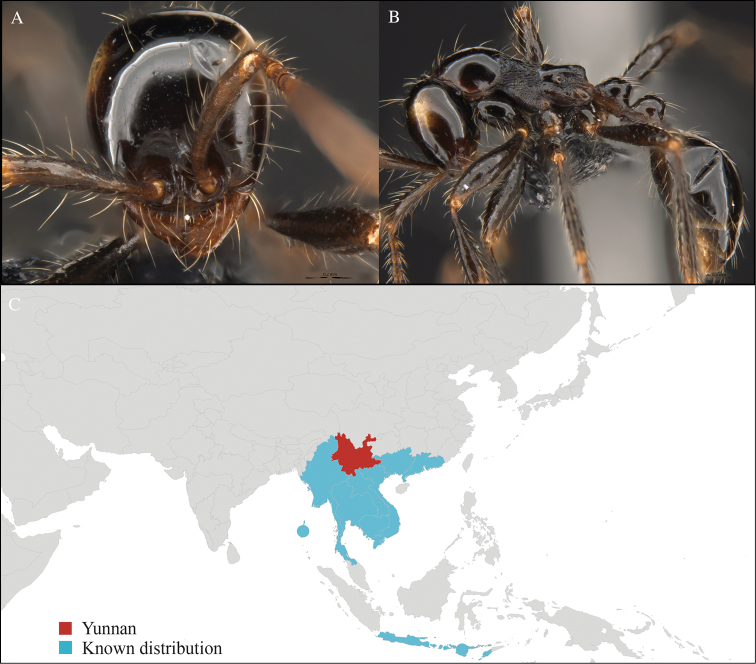
*Aenictus hodgsoni* worker, CASENT0716190. **A** Head in front view **B** Mesosoma in profile view **C** Global distribution map.

Widely distributed in the Indo-Malayan subregions (Figure 2C).

#### Taxonomic note.

*Aenictus hodgsoni* belongs to the *Aenictus laeviceps* s species group and can be easily identified with the identification key provided by Jaitrong and Yamane (2011).

#### Natural history.

*Aenictus hodgsoni* has been collected from leaf litter and foraging columns on the forest ground in secondary forest. This species has also been found from lowland to highland in varied forest types (hill evergreen forest, dry evergreen forest, evergreen rain forest, mixed deciduous forest, and savanna) (Jaitrong and Yamane 2011).

### Aenictus maneerati

Taxon classificationAnimaliaHymenopteraFormicidae

Jaitrong & Yamane, 2013

Figure 3

#### Material examined.

CHINA, Yunnan, Xishuangbanna: XTBG (21.916°N, 101.274°E), Secondary forest, 08.vi.2013, 1 worker, 615m, Winkler sifting, B. Guénard, B. Blanchard and C. Liu.

#### Distribution.

**Figure 3.**
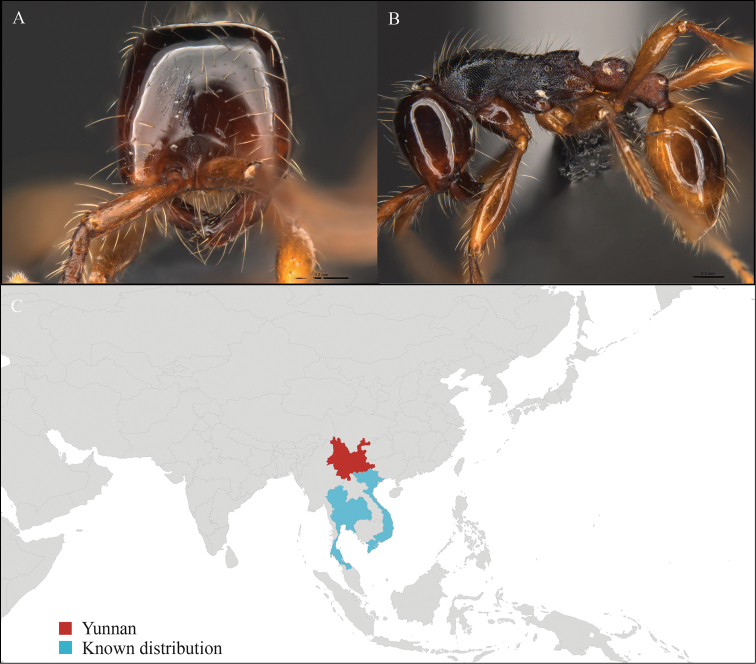
*Aenictus maneerati* worker, CASENT0717211. **A** Head in front view **B** Mesosoma in profile view **C** Global distribution map.

Yunnan (new record), Vietnam and Thailand (Figure 3C). Our material represents the northern-most record of *Aenictus hodgsoni*.

#### Taxonomic note.

*Aenictus hodgsoni* belongs to the *Aenictus ceylonicus* species group and can be easily identified with the identification key provided by Jaitrong and Yamane (2013).

#### Natural history.

Little is known about the bionomics of *Aenictus hodgsoni*. Before our survey, it has been only collected from primary forest (Jaitrong and Yamane 2013). We collected it from leaf litter in secondary forest.

### Aenictus paradentatus

Taxon classificationAnimaliaHymenopteraFormicidae

Jaitrong, Yamane & Tasen, 2012

Figure 4

#### Material examined.

CHINA, Yunnan, Xishuangbanna: XTBG (21.911°N, 101.281°E), Limestone forest, 06.vi.2013, 46 workers, 655m, Hand collection, B. Guénard, B. Blanchard and C. Liu.

#### Distribution.

**Figure 4.**
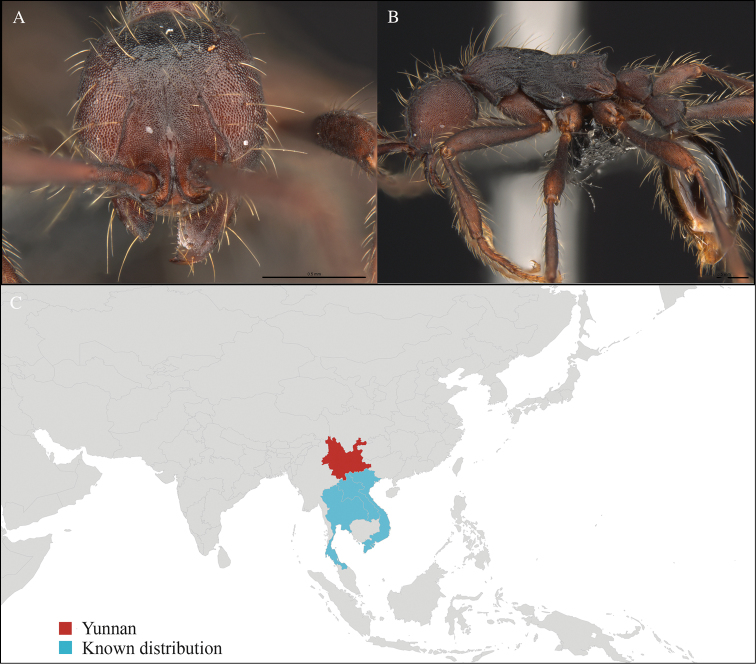
*Aenictus paradentatus* worker, CASENT0716195. **A** Head in front view **B** Mesosoma in profile view **C** Global distribution map.

Yunnan (new record), Vietnam, Laos, and Thailand (Figure 4C). This collection represents the northern-most record of *Aenictus paradentatus*.

#### Taxonomic note.

*Aenictus paradentatus* is very similar to *Aenictus dentatus* Forel, 1911, and can be easily identified with the key of Jaitrong et al. (2012).

#### Natural history.

*Aenictus paradentatus* has been collected from foraging columns on the ground in limestone forest, but was also reported to be found in other forest habitats, ranging from primary forest to disturbed forest (Jaitrong et al. 2012).

### Aenictus thailandianus

Taxon classificationAnimaliaHymenopteraFormicidae

Terayama & Kubota, 1993

Figure 5

#### Material examined.

CHINA, Yunnan, Xishuangbanna: XTBG (21.919°N, 101.274°E), Secondary forest, 11.vi.2013, 19 workers, 590m, Hand collection, B. Guénard, B. Blanchard and C. Liu; Man Sai village (21.857°N, 101.277°E), Rubber plantation, 12.vi.2013, 19 workers, 680m, Hand collection, B. Guénard, B. Blanchard and C. Liu; Man Sai village (21.857°N, 101.277°E), Rubber plantation, 12.vi.2013, 254 workers, 680m, Winkler sifting, B. Guénard, B. Blanchard and C. Liu.

#### Distribution.

**Figure 5.**
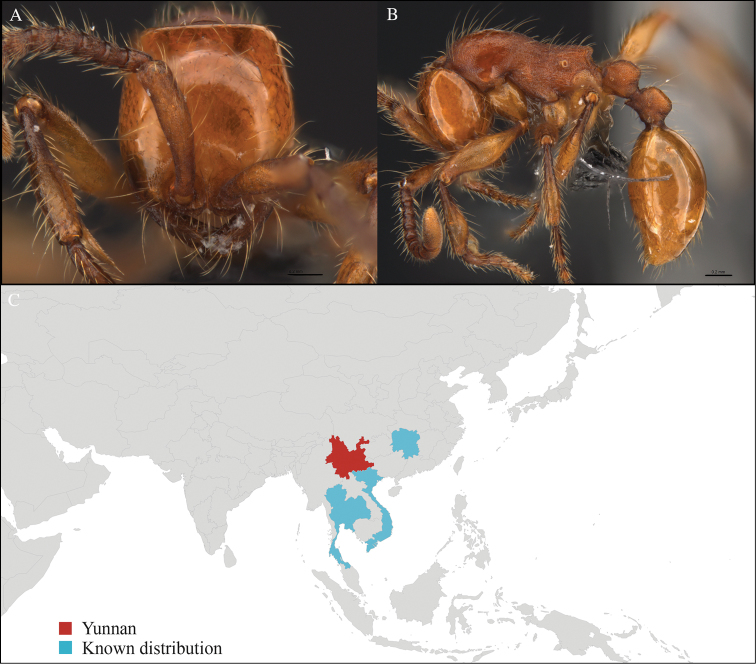
*Aenictus thailandianus* worker, CASENT0717202. **A** Head in front view **B** Mesosoma in profile view **C** Global distribution map.

Yunnan (new record), Hunan, Vietnam and Thailand (Figure 5C).

#### Taxonomic note.

*Aenictus thailandianus* belongs to the *Aenictus ceylonicus* species group and can be easily identified with the identification key presented by Jaitrong and Yamane (2013).

#### Natural history.

*Aenictus thailandianus* has only been found at higher elevations (1000–1500m) in primary and secondary forest (Jaitrong and Yamane 2013). We collected it from leaf litter and foraging columns on the ground in secondary forest and rubber plantations at lower elevations (under 1000m).

### Bannapone scrobiceps

Taxon classificationAnimaliaHymenopteraFormicidae

Guénard, Blanchard, Liu, Yang & Economo, 2013

Figure 6

#### Material examined.

CHINA, Yunnan, Xishuangbanna: XTBG (21.919°N, 101.272°E), Secondary forest, 05.vi.2013, 2 workers, 550m, Winkler sifting, B. Guénard, B. Blanchard and C. Liu.

#### Distribution.

**Figure 6.**
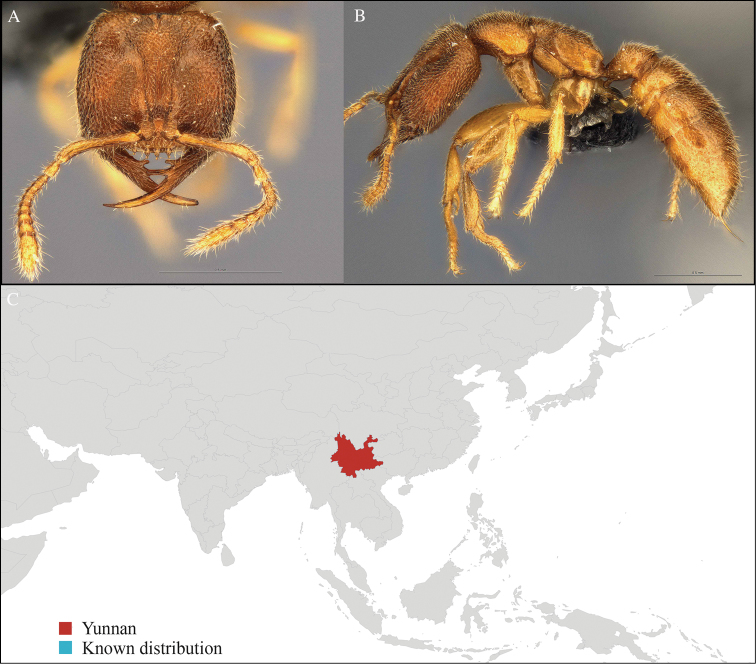
*Bannapone scrobiceps* worker, CASENT0339957. **A** Head in front view **B** Mesosoma in profile view **C** Global distribution map.

Yunnan (new record) (Figure 6C).

#### Taxonomic note.

*Bannapone scrobiceps* was described recently (Guénard et al. 2013).

#### Natural history.

Little is known about the bionomics of *Bannapone scrobiceps*. The species was collected from leaf litter in secondary forest located at 550 meters elevation (Guénard et al. 2013).

### Carebara melasolena

Taxon classificationAnimaliaHymenopteraFormicidae

(Zhou & Zheng, 1997)

Figure 7

#### Material examined.

CHINA, Yunnan, Xishuangbanna: Kilometer 55 station (21.960°N, 101.199°E), Rain forest, 10.vi.2013, 23 workers, 840m, Winkler sifting, B. Guénard, B. Blanchard and C. Liu.

#### Distribution.

**Figure 7.**
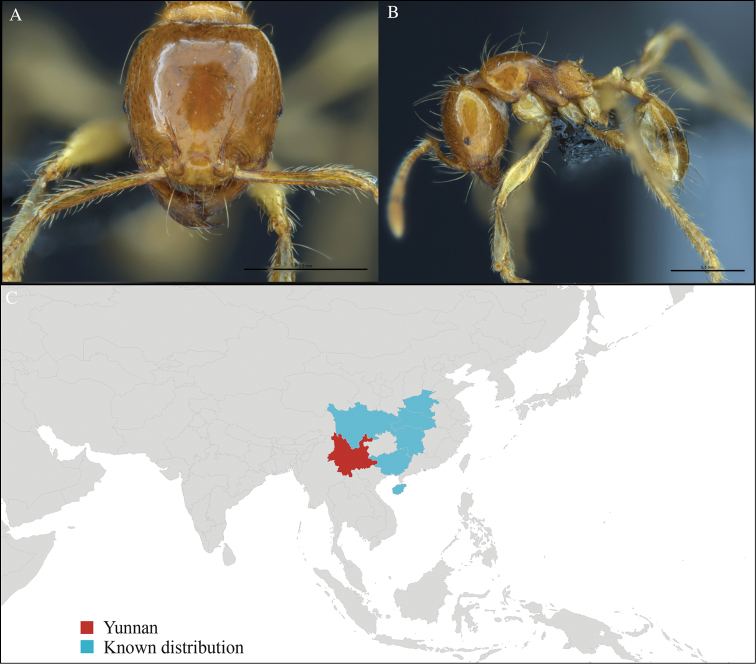
*Carebara melasolena* worker, CASENT0714818. **A** Head in front view **B** Mesosoma in profile view **C** Global distribution map.

Widely distributed in Middle and South China (Figure 7C).

#### Taxonomic note.

*Carebara melasolena* can be identified with the key provided by Zhou and Zheng (1997; treated as *Pheidologeton melasolenus*)

#### Natural history.

*Carebara melasolena* has been collected from leaf litter in primary forest.

### Discothyrea clavicornis

Taxon classificationAnimaliaHymenopteraFormicidae

Emery, 1897

Figure 8

#### Material examined.

CHINA, Yunnan, Xishuangbanna: Kilometer 55 station (21.962°N, 101.200°E), Rain forest, 10.vi.2013, 1 worker, 830m, Winkler sifting, B. Guénard, B. Blanchard and C. Liu; Kilometer 55 station (21.962°N, 101.200°E), Rain forest, 13.vi.2013, 8 workers, 805m, Winkler sifting, B. Guénard, B. Blanchard and C. Liu; Kilometer 55 station (21.962°N, 101.201°E), Rain forest, 13.vi.2013, 1 worker, 815m, Winkler sifting, B. Guénard, B. Blanchard and C. Liu; Kilometer 55 station (21.964°N, 101.202°E), Rain forest, 13.vi.2013, 3 workers, 820m, Winkler sifting, B. Guénard, B. Blanchard and C. Liu; Menglun town (21.932°N, 101.270°E), Rubber plantation, 09.vi.2013, 12 workers, 645m, Winkler sifting, B. Guénard, B. Blanchard and C. Liu; XTBG (21.919°N, 101.272°E), Secondary forest, 05.vi.2013, 6 worker, 550m, Winkler sifting, B. Guénard, B. Blanchard and C. Liu; XTBG (21.912°N, 101.285°E), Limestone forest, 06.vi.2013, 3 workers, 680m, Winkler sifting, B. Guénard, B. Blanchard and C. Liu; XTBG (21.919°N, 101.274°E), Limestone forest, 05.vi.2013, 15 workers, 552m, Winkler sifting, B. Guénard, B. Blanchard and C. Liu; XTBG (21.911°N, 101.284°E), Limestone forest, 06.vi.2013, 1 worker, 690m, Winkler sifting, B. Guénard, B. Blanchard and C. Liu; XTBG (21.918°N, 101.271°E), Rain forest, 05.vi.2013, 3 workers, 581m, Winkler sifting, B. Guénard, B. Blanchard and C. Liu; XTBG (21.911°N, 101.281°E), Limestone forest, 05.vi.2013, 1 worker, 650m, Winkler sifting, B. Guénard, B. Blanchard and C. Liu; XTBG (21.916°N, 101.274°E), Rain forest, 08.vi.2013, 3 workers, 615m, Winkler sifting, B. Guénard, B. Blanchard and C. Liu; XTBG (21.917°N, 101.274°E), Rain forest, 08.vi.2013, 2 workers, 625m, Winkler sifting, B. Guénard, B. Blanchard and C. Liu; Banna University construction site (21.922°N, 101.268°E), Rubber plantation, 14.vi.2013, 3 workers, 620m, Winkler sifting, B. Guénard, B. Blanchard and C. Liu.

#### Distribution.

**Figure 8.**
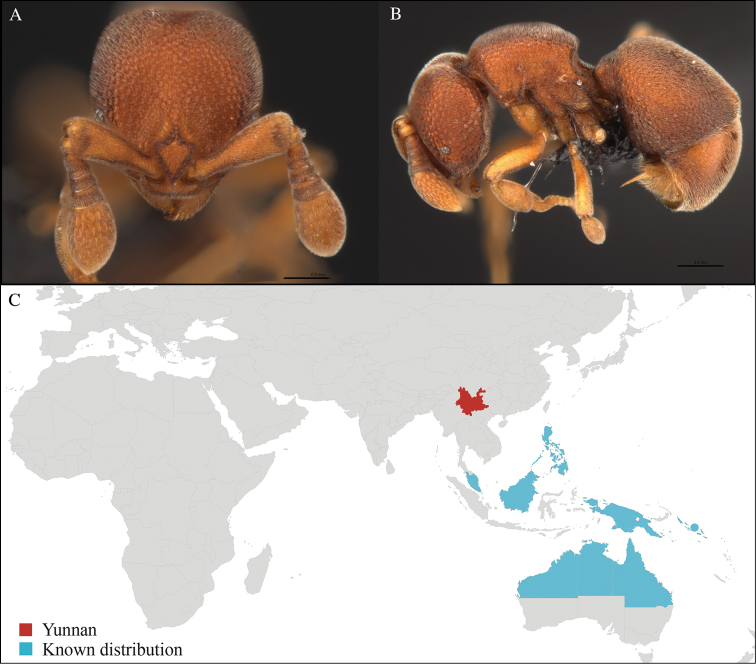
*Discothyrea clavicornis* worker, CASENT0735814. **A** Head in front view **B** Mesosoma in profile view **C** Global distribution map.

*Discothyrea clavicornis* is a very widespread and common species encountered throughout most of the Austral-Asian and Indo-Malayan subregions (Figure 8C). This new record represents an important extension of the northern range in the distribution of this species.

#### Taxonomic note.

There is no available key for *Discothyrea clavicornis*. Our identification is based on the original description (Emery 1897), comparison with reference material, and montage images of the holotype provided by AntWeb.

#### Natural history.

*Discothyrea clavicornis* has been collected from leaf litter in various habitats such as primary forest, limestone forest and rubber plantation.

### Discothyrea kamiteta

Taxon classificationAnimaliaHymenopteraFormicidae

Kubota & Terayama, 1999

Figure 9

#### Material examined.

CHINA, Yunnan, Xishuangbanna: Kilometer 55 station (21.963°N, 101.201°E), Rain forest, 13.vi.2013, 1 worker, 815m, Winkler sifting, B. Guénard, B. Blanchard and C. Liu; XTBG (21.911°N, 101.283°E), Limestone forest, 06.vi.2013, 1 worker, 675m, Winkler sifting, B. Guénard, B. Blanchard and C. Liu; XTBG (21.917°N, 101.274°E), Secondary forest, 08.vi.2013, 1 worker, 625m, Winkler sifting, B. Guénard, B. Blanchard and C. Liu; Man Sai village (21.858°N, 101.276°E), Secondary forest, 12.vi.2013, 1 worker, 690m, Winkler sifting, B. Guénard, B. Blanchard and C. Liu.

#### Distribution.

**Figure 9.**
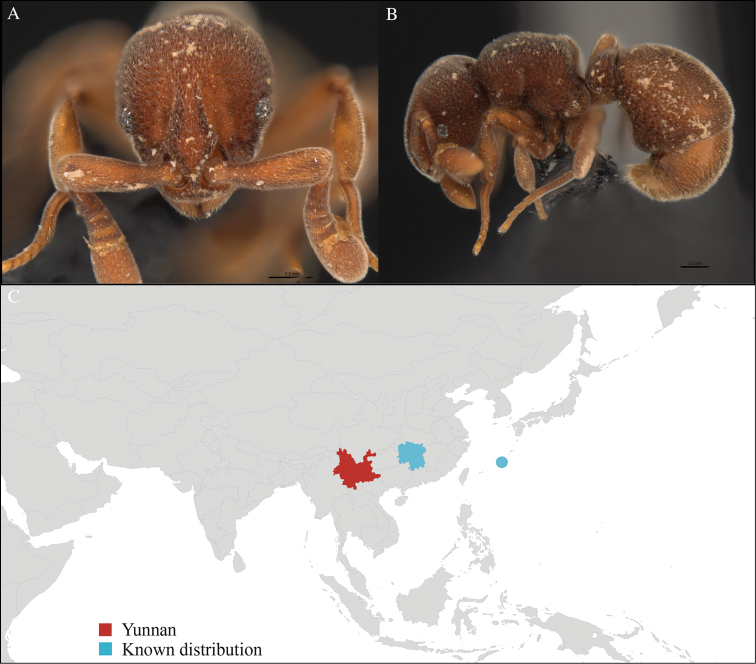
*Discothyrea kamiteta* worker, CASENT0717828. **A** Head in front view **B** Mesosoma in profile view **C** Global distribution map.

Yunnan (new record), Hunan, Okinawa (Figure 9C). This new record represents an important western-most extension in the known distribution of this species.

#### Taxonomic note.

*Discothyrea kamiteta* is very similar to the recently described *Discothyrea banna* Xu, Burwell & Nakamura, 2014. Both species seem to be very close morphologically, and their separation is based on minor differences, which could also be attributed to intraspecific variation. The identification is based on the original description of *Discothyrea kamiteta*, comparison with *Discothyrea kamiteta* material from the type locality (Okinawa), and Xu’s key (Xu et al. 2014)

#### Natural history.

*Discothyrea kamiteta* has been collected from leaf litter in various habitats, such as primary forest, limestone forest and secondary forest.

### Dolichoderus laotius

Taxon classificationAnimaliaHymenopteraFormicidae

Santschi, 1920

Figure 10

#### Material examined.

CHINA, Yunnan, Xishuangbanna: Man Sai village (21.858°N, 101.276°E), Secondary forest, 12.vi.2013, 5 worker, 690m, Hand collection, B. Guénard, B. Blanchard and C. Liu.

#### Distribution.

**Figure 10.**
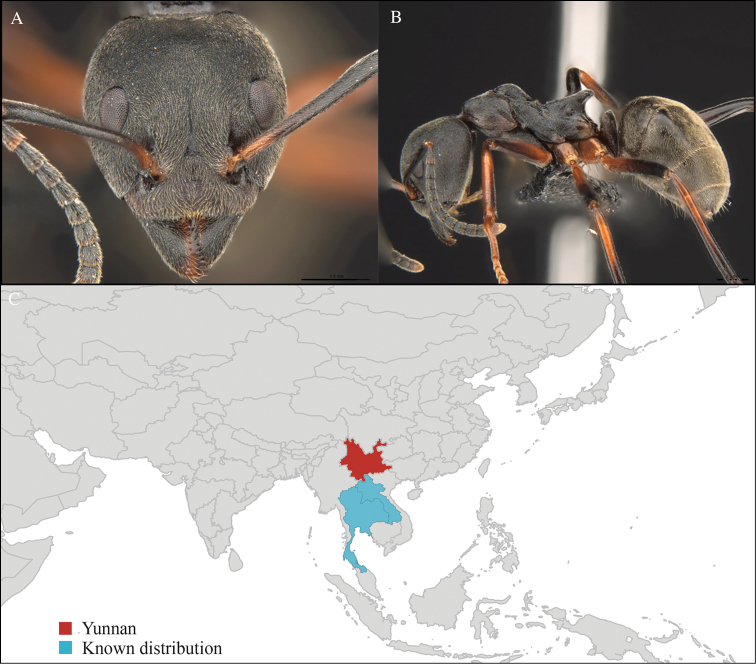
*Dolichoderus laotius* worker, CASENT0716164. **A** Head in front view **B** Alitrunk in profile view **C** Global distribution map.

Yunnan (new record), Laos, Thailand (Figure 10C). This collection represents the northern-most record of *Dolichoderus laotius*.

#### Taxonomic note.

There is no available key for the genus in the region. Our identification is based on the description provided by Dill et al. (2002).

#### Natural history.

Little is known about the bionomics of *Dolichoderus laotius*. This species has been collected on a tree trunk in secondary forest.

### Echinopla cherapunjiensis

Taxon classificationAnimaliaHymenopteraFormicidae

Bharti & Gul, 2012

Figure 11

#### Material examined.

CHINA, Yunnan, Xishuangbanna: XTBG (21.919°N, 101.273°E), Secondary forest, 08.vi.2013, 1 worker, 615m, Hand collection, B. Guénard, B. Blanchard and C. Liu.

#### Distribution.

**Figure 11.**
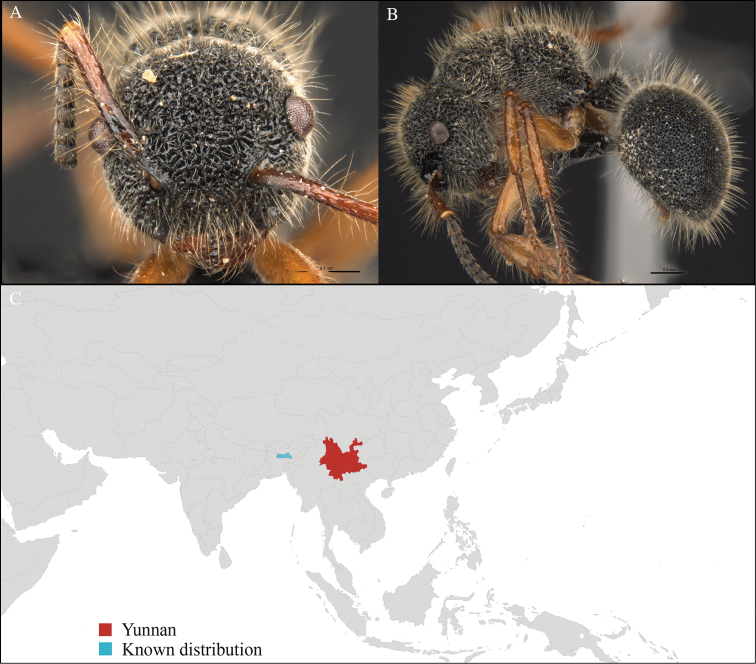
*Echinopla cherapunjiensis* worker, CASENT0716524. **A** Head in front view **B** Mesosoma in profile view **C** Global distribution map.

Yunnan (new record) and Meghalaya (Figure 11C). This new record represents an important northern and western extension in the distribution of *Echinopla cherapunjiensis*.

#### Taxonomic note.

There is no available key for this genus. Identification is based on the original description (Bharti and Gul 2012).

#### Natural history.

Little is known about the bionomics of *Echinopla cherapunjiensis*. This species has been collected on a tree trunk in secondary forest.

### Gesomyrmex kalshoveni

Taxon classificationAnimaliaHymenopteraFormicidae

Wheeler, 1929

Figure 12

#### Material examined.

CHINA, Yunnan, Xishuangbanna: XTBG (21.925°N, 101.270°E), Forest fragment, 08.vi.2013, 1 worker, 615m, Hand collection, B. Guénard, B. Blanchard and C. Liu.

#### Distribution.

**Figure 12.**
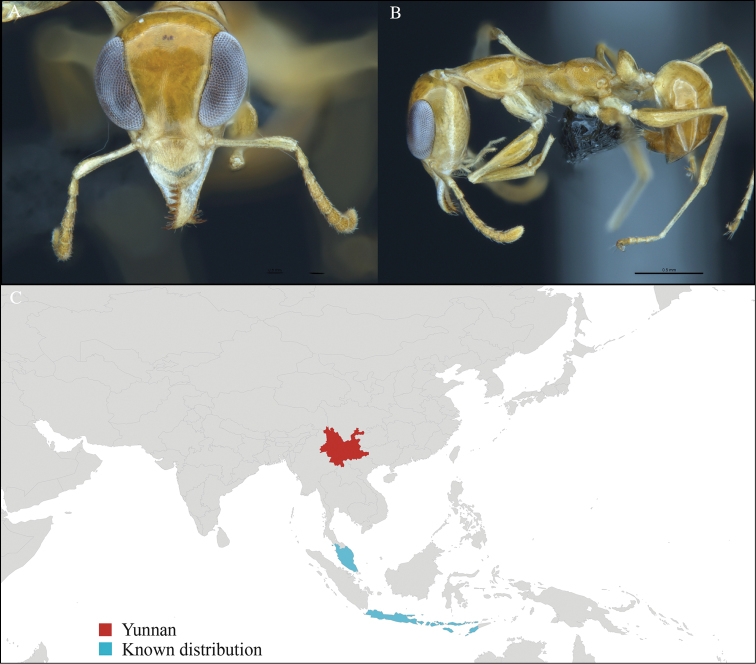
*Gesomyrmex kalshoveni* worker, CASENT0716525. **A** Head in front view **B** Mesosoma in profile view **C** Global distribution map.

Yunnan (new record), Malaysia and Indonesia (Figure 12C). This new record represents an important extension in the northern range of the distribution of this species and the first occurrence of the genus *Gesomyrmex* from Yunnan province.

#### Taxonomic note.

There is no available key for this genus. The identification is based on the original description (Wheeler 1929) and comparison with reference material from Borneo. Identification in *Gesomyrmex* is generally very difficult due to the high degree of worker polymorphism. However, our single specimen is a minor worker and fits the minor workers of *Gesomyrmex kalshoveni* very well.

#### Natural history.

Little is known about the bionomics of *Gesomyrmex kalshoveni*. It has been collected from a small branch of a tree on the side of road.

### Gnamptogenys costata

Taxon classificationAnimaliaHymenopteraFormicidae

(Emery, 1889)

Figure 13

#### Material examined.

CHINA, Yunnan, Xishuangbanna: XTBG (21.911°N, 101.281°E), Limestone forest, 06.vi.2013, 1 worker, 655m, Hand collection, B. Guénard, B. Blanchard and C. Liu; XTBG (21.919°N, 101.274°E), Rain forest, 08.vi.2013, 2 workers, 615m, Hand collection, B. Guénard, B. Blanchard and C. Liu

#### Distribution.

**Figure 13.**
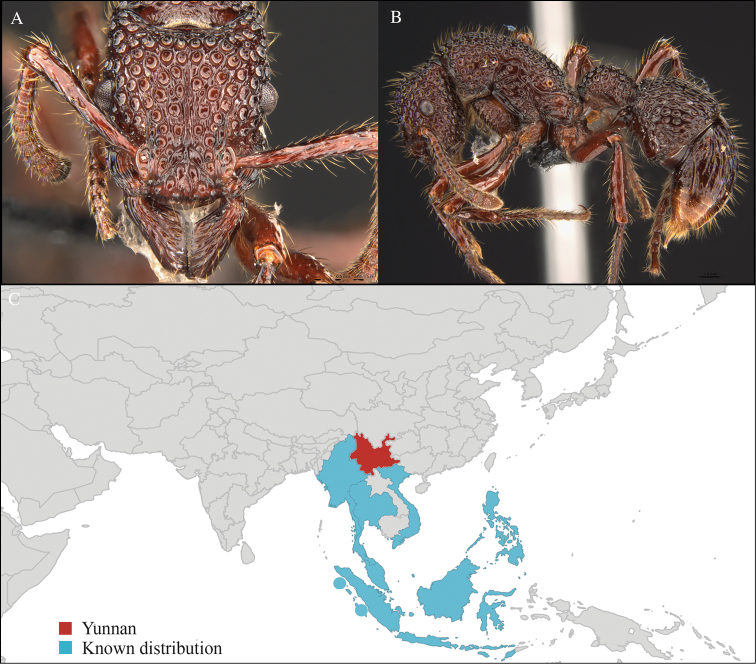
*Gnamptogenys costata* worker, CASENT0715692. **A** Head in front view **B** Mesosoma in profile view **C** Global distribution map.

Widely distributed in the Austral-Asian and Indo-Malayan subregions (Figure 13C).

#### Taxonomic note.

The identification is based on the key provided by Lattke (2004). The material from Yunnan shows some minor variation in the development of gastral sculpture, which we consider as geographic, intraspecific variation.

#### Natural history.

*Gnamptogenys costata* has been collected from foraging columns on the ground in rain forest and limestone forest.

### Gnamptogenys treta

Taxon classificationAnimaliaHymenopteraFormicidae

Lattke, 2004

Figure 14

#### Material examined.

CHINA, Yunnan, Xishuangbanna: XTBG (21.912°N, 101.285°E), Limestone forest, 06.vi.2013, 1 worker, 655m, Winkler sifting, B. Guénard, B. Blanchard and C. Liu; “Holy Hills” (21.920°N, 101.240°E), Rain forest, 07.vi.2013, 1 worker, 665m, Winkler sifting, B. Guénard, B. Blanchard and C. Liu; Menglun town (21.932°N, 101.271°E), Rubber plantation, 09.vi.2013, 7 workers, 645m, Winkler sifting, B. Guénard, B. Blanchard and C. Liu; Man Sai village (21.858°N, 101.277°E), Secondary forest, 12.vi.2013, 2 workers, 690m, Winkler sifting, B. Guénard, B. Blanchard and C. Liu; Kilometer 55 station (21.962°N, 101.200°E), Rain forest, 13.vi.2013, 10 workers, 865m, Winkler sifting, B. Guénard, B. Blanchard and C. Liu

#### Distribution.

**Figure 14.**
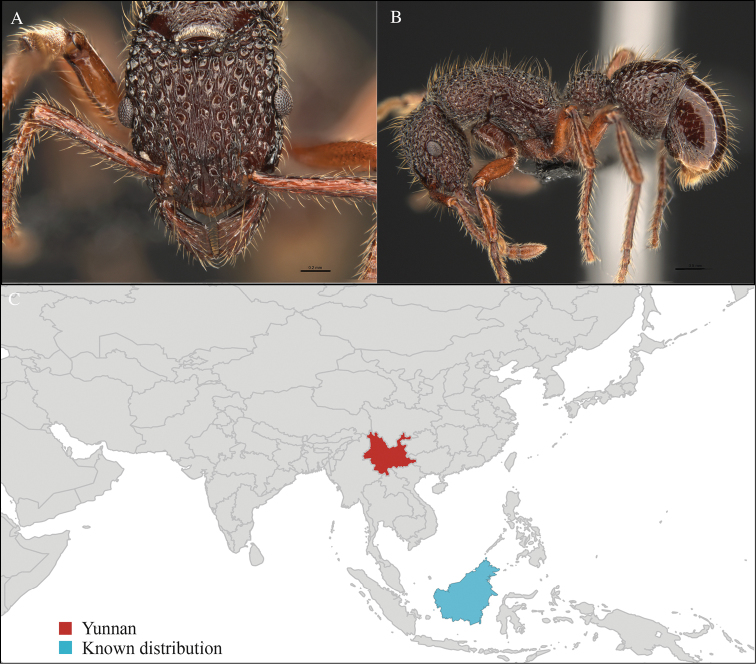
*Gnamptogenys treta* worker, CASENT0715166. **A** Head in front view **B** Mesosoma in profile view **C** Global distribution map.

Known from Yunnan (new record) and Borneo (Figure 14C). This collection represents the northern-most record in the distribution of *Gnamptogenys treta*.

#### Taxonomic note.

The identification is based on the key provided by Lattke (2004). Our material fits the holotype very well, except for the shape of the ventral process of the petiole, which is more rectangular in the material from Yunnan, whereas in the material from Borneo it is more triangular. Since this is the only difference we were able to observe, we treat it as intraspecific variation.

#### Natural history.

*Gnamptogenys treta* has been collected from the leaf litter in rain forest, secondary forest and limestone forest and rubber plantation.

### Myrmecina curvispina

Taxon classificationAnimaliaHymenopteraFormicidae

Zhou, Huang & Ma L., 2008

Figure 15

#### Material examined.

CHINA, Yunnan, Xishuangbanna: “Holy Hills” (21.920°N, 101.240°E), Rain forest, 07.vi.2013, 1 worker, 655m, Winkler sifting, B. Guénard, B. Blanchard and C. Liu; “Holy Hills” (21.919°N, 101.239°E), Rain forest, 07.vi.2013, 1 worker, 670m, Winkler sifting, B. Guénard, B. Blanchard and C. Liu; Kilometer 55 station (21.961°N, 101.200°E), Rain forest, 10.vi.2013, 1 worker, 820m, Winkler sifting, B. Guénard, B. Blanchard and C. Liu; Kilometer 55 station (21.966°N, 101.203°E), Secondary forest, 13.vi.2013, 12 workers, 840m, Winkler sifting, B. Guénard, B. Blanchard and C. Liu; Kilometer 55 station (21.963°N, 101.201°E), Rain forest, 13.vi.2013, 14 workers, 815m, Winkler sifting, B. Guénard, B. Blanchard and C. Liu; Man Sai village (21.858°N, 101.277°E), Rubber plantation, 12.vi.2013, 1 worker, 705m, Winkler sifting, B. Guénard, B. Blanchard and C. Liu;: Man Sai village (21.907°N, 101.273°E), Rubber plantation, 12.vi.2013, 2 workers, 635m, Winkler sifting, B. Guénard, B. Blanchard and C. Liu;: Man Sai village (21.858°N, 101.277°E), Secondary forest, 12.vi.2013, 2 workers, 685m, Winkler sifting, B. Guénard, B. Blanchard and C. Liu; Man Sai village (21.858°N, 101.276°E), Secondary forest, 12.vi.2013, 3 workers, 690m, Winkler sifting, B. Guénard, B. Blanchard and C. Liu; Man Sai village (21.860°N, 101.278°E), Secondary forest, 12.vi.2013, 2 workers, 680m, Winkler sifting, B. Guénard, B. Blanchard and C. Liu; Menglun town (21.932°N, 101.271°E), Rubber plantation, 09.vi.2013, 3 workers, 640m, Winkler sifting, B. Guénard, B. Blanchard and C. Liu; XTBG (21.919°N, 101.272°E), Secondary forest, 05.vi.2013, 1 worker, 550m, Winkler sifting, B. Guénard, B. Blanchard and C. Liu; XTBG (21.912°N, 101.182°E), Limestone forest, 06.vi.2013, 9 workers, 640m, Winkler sifting, B. Guénard, B. Blanchard and C. Liu; Banna University construction site (21.888°N, 101.266°E), Rubber Plantation, 14.vi.2013, 7 workers, 600m, Winkler sifting, B. Guénard, B. Blanchard and C. Liu; Banna University construction site (21.889°N, 101.267°E), Rubber Plantation, 14.vi.2013, 3 workers, 630m, Winkler sifting, B. Guénard, B. Blanchard and C. Liu; Banna University construction site (21.890°N, 101.267°E), Rubber Plantation, 14.vi.2013, 2 workers, 620m, Winkler sifting, B. Guénard, B. Blanchard and C. Liu.

#### Distribution.

**Figure 15.**
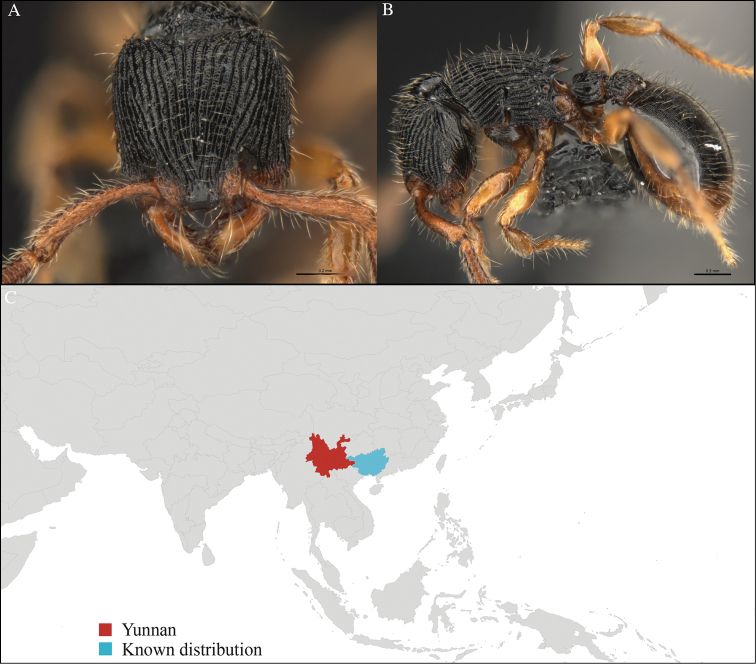
*Myrmecina curvispina* worker, CASENT0713308. **A** Head in front view **B** Mesosoma in profile view **C** Global distribution map.

Yunnan (new record) and Guangxi (Figure 15C). This new record represents the western-most occurrence in the distribution of *Myrmecina curvispina*.

#### Taxonomic note.

The identification is based on the original description and the identification key given by Zhou et al. (2008).

#### Natural history.

*Myrmecina curvispina* has been collected from the leaf litter of various habitats such as rain forest, secondary forest and rubber plantation.

### Myrmecina guangxiensis

Taxon classificationAnimaliaHymenopteraFormicidae

Zhou, 2001

Figure 16

#### Material examined.

CHINA, Yunnan, Xishuangbanna: XTBG (21.912°N, 101.285°E), Secondary forest, 05.vi.2013, 4 workers, 552m, Winkler sifting, B. Guénard, B. Blanchard and C. Liu; XTBG (21.919°N, 101.274°E), Limestone forest, 06.vi.2013, 1 worker, 680m, Winkler sifting, B. Guénard, B. Blanchard and C. Liu; XTBG (21.911°N, 101.281°E), Limestone forest, 06.vi.2013, 2 workers, 650m, Winkler sifting, B. Guénard, B. Blanchard and C. Liu; Kilometer 55 station (21.960°N, 101.199°E), Rain forest, 10.vi.2013, 1 worker, 840m, Winkler sifting, B. Guénard, B. Blanchard and C. Liu; Kilometer 55 station (21.963°N, 101.201°E), Rain forest, 13.vi.2013, 9 workers, 815m, Winkler sifting, B. Guénard, B. Blanchard and C. Liu.

#### Distribution.

**Figure 16.**
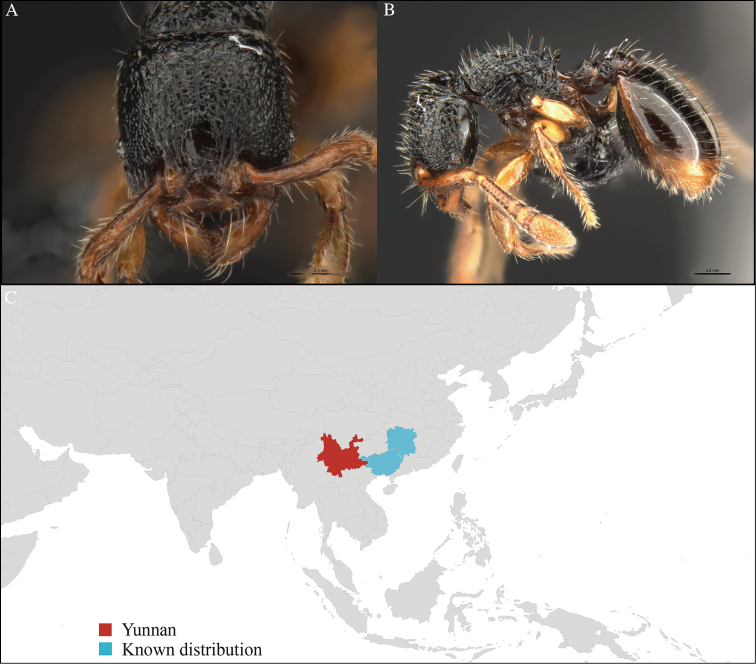
*Myrmecina guangxiensis* worker, CASENT0713314. **A** Head in front view **B** Mesosoma in profile view **C** Global distribution map.

Yunnan (new record), Guangxi and Hunan (Figure 16C). This new record represents the western-most occurrence in the distribution of *Myrmecina guangxiensis*.

#### Taxonomic note.

Identification is based on the key provided by Zhou et al. (2008).

#### Natural history.

*Myrmecina guangxiensis* has been collected from leaf litter in rain forest, secondary forest and limestone forest.

### Odontoponera denticulata

Taxon classificationAnimaliaHymenopteraFormicidae

(Smith, 1858)

Figure 17

#### Material examined.

CHINA, Yunnan, Xishuangbanna: “Holy Hills” (21.920°N, 101.240°E), Secondary forest, 07.vi.2013, 1 worker, 655m, Winkler sifting, B. Guénard, B. Blanchard and C. Liu; “Holy Hills” (21.920°N, 101.239°E), Secondary forest, 07.vi.2013, 2 workers, 665m, Winkler sifting, B. Guénard, B. Blanchard and C. Liu; Kilometer 55 station (21.966°N, 101.203°E), Secondary forest, 13.vi.2013, 1 worker, 825m, Winkler sifting, B. Guénard, B. Blanchard and C. Liu; Kilometer 55 station (21.966°N, 101.203°E), Secondary forest, 13.vi.2013, 1 worker, 840m, Winkler sifting, B. Guénard, B. Blanchard and C. Liu; Kilometer 55 station (21.962°N, 101.200°E), Rain forest, 13.vi.2013, 3 workers, 805m, Winkler sifting, B. Guénard, B. Blanchard and C. Liu; Kilometer 55 station (21.963°N, 101.201°E), Rain forest, 13.vi.2013, 2 workers, 815m, Winkler sifting, B. Guénard, B. Blanchard and C. Liu; Kilometer 55 station (21.964°N, 101.202°E), Rain forest, 13.vi.2013, 1 worker, 820m, Winkler sifting, B. Guénard, B. Blanchard and C. Liu; Man Sai village (21.907°N, 101.273°E), Rubber plantation, 12.vi.2013, 1 worker, 635m, Winkler sifting, B. Guénard, B. Blanchard and C. Liu; Man Sai village (21.860°N, 101.278°E), Rubber plantation, 12.vi.2013, 1 worker, 710m, Winkler sifting, B. Guénard, B. Blanchard and C. Liu; Man Sai village (21.858°N, 101.276°E), Secondary forest, 12.vi.2013, 1 worker, 685m, Winkler sifting, B. Guénard, B. Blanchard and C. Liu; Man Sai village (21.860°N, 101.278°E), Secondary forest, 12.vi.2013, 1 worker, 680m, Winkler sifting, B. Guénard, B. Blanchard and C. Liu; Menglun town (21.933°N, 101.269°E), Rubber plantation, 09.vi.2013, 1 worker, 655m, Winkler sifting, B. Guénard, B. Blanchard and C. Liu; Menglun town (21.932°N, 101.271°E), Rubber plantation, 09.vi.2013, 5 workers, 640m, Winkler sifting, B. Guénard, B. Blanchard and C. Liu; Menglun town (21.932°N, 101.270°E), Rubber plantation, 09.vi.2013, 2 workers, 645m, Winkler sifting, B. Guénard, B. Blanchard and C. Liu; Menglun town (21.932°N, 101.269°E), Rubber plantation, 09.vi.2013, 1 worker, 645m, Winkler sifting, B. Guénard, B. Blanchard and C. Liu; XTBG (21.924°N, 101.268°E), Rubber plantation, 06.vi.2013, 3 workers, 571m, Winkler sifting, B. Guénard, B. Blanchard and C. Liu; XTBG (21.919°N, 101.272°E), Secondary forest, 05.vi.2013, 1 worker, 550m, Winkler sifting, B. Guénard, B. Blanchard and C. Liu; XTBG (21.911°N, 101.284°E), Limestone forest, 06.vi.2013, 3 workers, 690m, Winkler sifting, B. Guénard, B. Blanchard and C. Liu; XTBG (21.912°N, 101.282°E), Limestone forest, 05.vi.2013, 1 worker, 640m, Winkler sifting, B. Guénard, B. Blanchard and C. Liu; XTBG (21.917°N, 101.274°E), Secondary forest, 08.vi.2013, 1 worker, 625m, Winkler sifting, B. Guénard, B. Blanchard and C. Liu; Banna University construction site (21.889°N, 101.267°E), Rubber Plantation, 14.vi.2013, 4 workers, 630m, Winkler sifting, B. Guénard, B. Blanchard and C. Liu; Banna University construction site (21.888°N, 101.266°E), Rubber Plantation, 14.vi.2013, 2 workers, 600m, Winkler sifting, B. Guénard, B. Blanchard and C. Liu; Banna University construction site (21.888°N, 101.266°E), Rubber Plantation, 14.vi.2013, 3 workers, 620m, Winkler sifting, B. Guénard, B. Blanchard and C. Liu; Banna University construction site (21.889°N, 101.267°E), Rubber Plantation, 14.vi.2013, 3 workers, 630m, Winkler sifting, B. Guénard, B. Blanchard and C. Liu; Banna University construction site (21.922°N, 101.268°E), Rubber Plantation, 14.vi.2013, 4 workers, 620m, Winkler sifting, B. Guénard, B. Blanchard and C. Liu; Banna University construction site (21.890°N, 101.267°E), Rubber Plantation, 14.vi.2013, 2 workers, 620m, Winkler sifting, B. Guénard, B. Blanchard and C. Liu.

#### Distribution.

**Figure 17.**
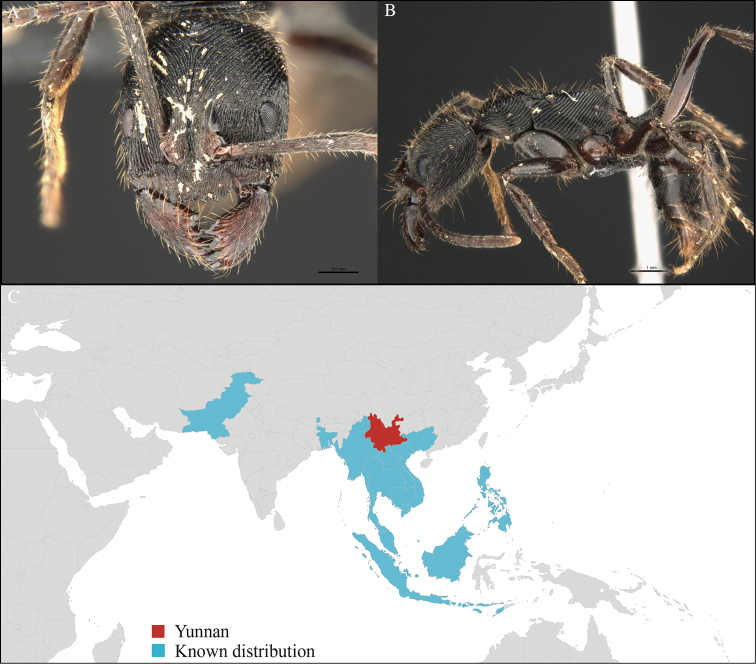
*Odontoponera denticulata* worker, CASENT0717236. **A** Head in front view **B** Mesosoma in profile view **C** Global distribution map.

Widely distributed in the Indo-Malayan subregion (Figure 17C).

#### Taxonomic note.

The identification of our material is based on Yamane’s (2009) redescription of *Odontoponera denticulata*.

#### Natural history.

*Odontoponera denticulata* has been collected from the leaf litter in various habitats such as rain forest, secondary forest, limestone forest and rubber plantation.

### Pheidole hongkongensis

Taxon classificationAnimaliaHymenopteraFormicidae

Wheeler, 1928

Figure 18

#### Material examined.

CHINA, Yunnan, Xishuangbanna: XTBG (21.919°N, 101.272°E), Secondary forest, 05.vi.2013, 1 worker, 550m, Winkler sifting, B. Guénard, B. Blanchard and C. Liu; XTBG (21.919°N, 101.274°E), Secondary forest, 05.vi.2013, 3 workers, 1 Soldier, 552m, Winkler sifting, B. Guénard, B. Blanchard and C. Liu; XTBG (21.924°N, 101.268°E), Secondary forest, 05.vi.2013, 2 workers, 571m, Winkler sifting, B. Guénard, B. Blanchard and C. Liu; XTBG (21.918°N, 101.271°E), Secondary forest, 05.vi.2013, 1 worker, 581m, Winkler sifting, B. Guénard, B. Blanchard and C. Liu; XTBG (21.912°N, 101.285°E), Limestone forest, 06.vi.2013, 49 workers, 680m, Winkler sifting, B. Guénard, B. Blanchard and C. Liu; XTBG (21.912°N, 101.282°E), Limestone forest, 06.vi.2013, 6 workers, 1 Soldier, 640m, Winkler sifting, B. Guénard, B. Blanchard and C. Liu; Menglun town (21.933°N, 101.269°E), Rubber plantation, 09.vi.2013, 3 workers, 655m, Winkler sifting, B. Guénard, B. Blanchard and C. Liu; Man Sai village (21.858°N, 101.276°E), Secondary forest, 12.vi.2013, 20 workers, 685m, Winkler sifting, B. Guénard, B. Blanchard and C. Liu; Man Sai village (21.860°N, 101.278°E), Secondary forest, 12.vi.2013, 1 worker, 680m, Winkler sifting, B. Guénard, B. Blanchard and C. Liu; Man Sai village (21.907°N, 101.273°E), Rubber plantation, 12.vi.2013, 3 workers, 635m, Winkler sifting, B. Guénard, B. Blanchard and C. Liu; Kilometer 55 station (21.962°N, 101.200°E), Rain forest, 13.vi.2013, 5 workers, 1 Soldier, 805m, Winkler sifting, B. Guénard, B. Blanchard and C. Liu; Banna University construction site (21.922°N, 101.268°E), Rubber Plantation, 14.vi.2013, 1 worker, 629m, Winkler sifting, B. Guénard, B. Blanchard and C. Liu.

#### Distribution.

**Figure 18.**
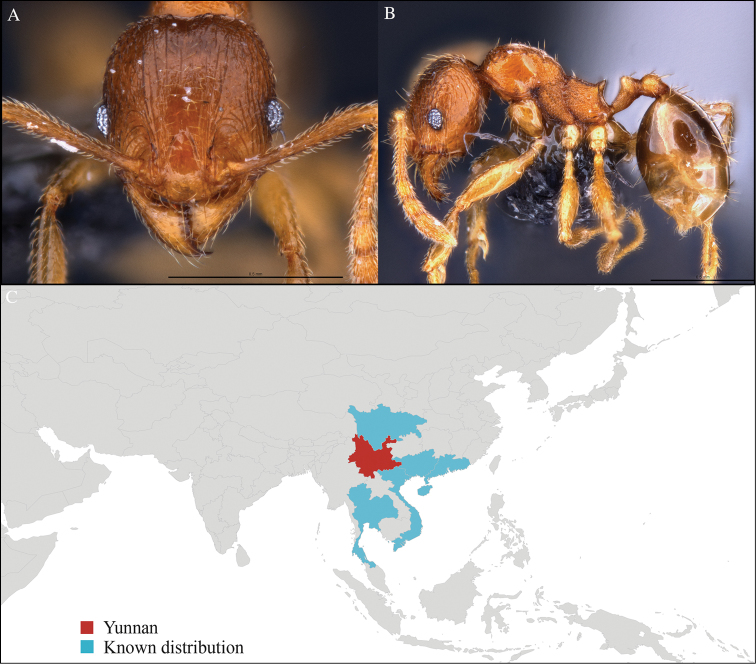
*Pheidole hongkongensis* worker, CASENT0714788. **A** Head in front view **B** Mesosoma in profile view **C** Global distribution map.

South China, Vietnam and Thailand (Figure 18C).

#### Taxonomic note.

*Pheidole hongkongensis* can be identified with the identification key to northern Vietnamese *Pheidole* published by Eguchi (2008).

#### Natural history.

*Pheidole hongkongensis* has been collected from leaf litter in secondary forest, limestone forest and rubber plantations. It has also been reported inhabiting the soil of woody gardens, forest edges and open areas (Eguchi 2008).

### Pheidole plagiaria

Taxon classificationAnimaliaHymenopteraFormicidae

Smith, 1860

Figure 19

#### Material examined.

CHINA, Yunnan, Xishuangbanna: XTBG (21.912°N, 101.282°E), Limestone forest, 06.vi.2013, 2 workers, 640m, Winkler sifting, B. Guénard, B. Blanchard and C. Liu; Menglun town (21.933°N, 101.269°E), Rubber Plantation, 09.vi.2013, 1 worker, 655m, Winkler sifting, B. Guénard, B. Blanchard and C. Liu; Kilometer 55 station (21.960°N, 101.199°E), Rain forest, 10.vi.2013, 13 workers, 840m, Winkler sifting, B. Guénard, B. Blanchard and C. Liu; Kilometer 55 station (21.962°N, 101.200°E), Rain forest, 10.vi.2013, 2 workers, 830m, Winkler sifting, B. Guénard, B. Blanchard and C. Liu.

#### Distribution.

**Figure 19.**
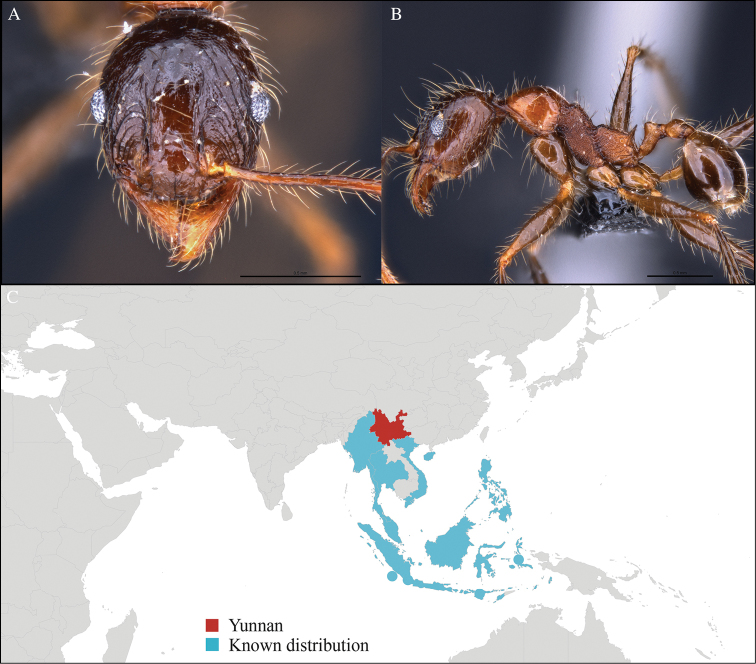
*Pheidole plagiaria* worker, CASENT0713421. **A** Head in front view **B** Mesosoma in profile view **C** Global distribution map.

Widely distributed in the Australasian and Indo-Malayan subregions (Figure 19C).

#### Taxonomic note.

*Pheidole plagiaria* can be identified with the identification key to Northern Vietnamese *Pheidole* provided by Eguchi (2008).

#### Natural history.

*Pheidole plagiaria* has been collected from leaf litter from rain forest, limestone forest and rubber plantation. It has also been reported inhabiting in the soil of forest edge and open land (Eguchi 2008).

### Pheidole planifrons

Taxon classificationAnimaliaHymenopteraFormicidae

Santschi, 1920

Figure 20

#### Material examined.

CHINA, Yunnan, Xishuangbanna: XTBG (21.919°N, 101.274°E), Secondary forest, 05.vi.2013, 2 workers, 552m, Winkler sifting, B. Guénard, B. Blanchard and C. Liu; XTBG (21.924°N, 101.268°E), Rubber plantation, 05.vi.2013, 3 workers, 571m, Winkler sifting, B. Guénard, B. Blanchard and C. Liu; XTBG (21.911°N, 101.283°E), Limestone forest, 06.vi.2013, 2 workers, 675m, Winkler sifting, B. Guénard, B. Blanchard and C. Liu; XTBG (21.912°N, 101.282°E), Limestone forest, 06.vi.2013, 4 workers, 640m, Winkler sifting, B. Guénard, B. Blanchard and C. Liu; XTBG (21.911°N, 101.281°E), Limestone forest, 06.vi.2013, 2 workers, 650m, Winkler sifting, B. Guénard, B. Blanchard and C. Liu; XTBG (21.916°N, 101.274°E), Limestone forest, 08.vi.2013, 2 workers, 615m, Winkler sifting, B. Guénard, B. Blanchard and C. Liu; Menglun town (21.932°N, 101.271°E), Rubber plantation, 09.vi.2013, 1 worker, 640m, Winkler sifting, B. Guénard, B. Blanchard and C. Liu; Man Sai village (21.858°N, 101.277°E), Rubber plantation, 12.vi.2013, 4 workers, 705m, Winkler sifting, B. Guénard, B. Blanchard and C. Liu; Man Sai village (21.857°N, 101.277°E), Rubber plantation, 12.vi.2013, 1 worker, 710m, Winkler sifting, B. Guénard, B. Blanchard and C. Liu; Man Sai village (21.907°N, 101.273°E), Rubber plantation, 12.vi.2013, 2 workers, 635m, Winkler sifting, B. Guénard, B. Blanchard and C. Liu; Man Sai village (21.858°N, 101.277°E), Secondary forest, 12.vi.2013, 2 workers, 685m, Winkler sifting, B. Guénard, B. Blanchard and C. Liu; Man Sai village (21.858°N, 101.276°E), Secondary forest, 12.vi.2013, 9 workers, 690m, Winkler sifting, B. Guénard, B. Blanchard and C. Liu; Man Sai village (21.860°N, 101.273°E), Secondary forest, 12.vi.2013, 1 worker, 680m, Winkler sifting, B. Guénard, B. Blanchard and C. Liu; Kilometer 55 station (21.966°N, 101.203°E), Rain forest, 13.vi.2013, 3 workers, 840m, Winkler sifting, B. Guénard, B. Blanchard and C. Liu; Banna University construction site (21.889°N, 101.267°E), Rubber Plantation, 14.vi.2013, 33 workers, 630m, Winkler sifting, B. Guénard, B. Blanchard and C. Liu; Banna University construction site (21.922°N, 101.268°E), Rubber Plantation, 14.vi.2013, 2 workers, 620m, Winkler sifting, B. Guénard, B. Blanchard and C. Liu.

#### Distribution.

**Figure 20.**
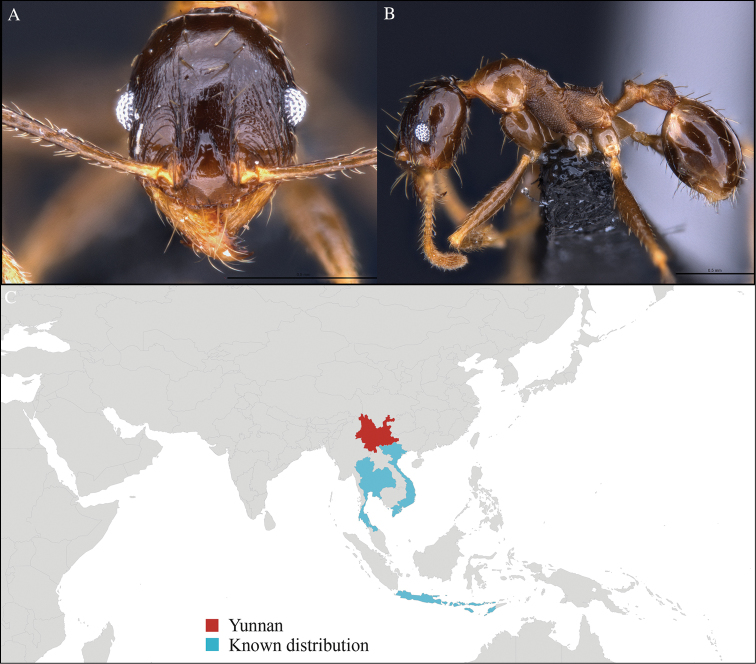
*Pheidole planifrons* worker, CASENT0713099. **A** Head in front view **B** Mesosoma in profile view **C** Global distribution map.

Yunnan (new record), Vietnam, Thailand and Java (Figure 20C). This new record represents the northern-most occurrence in the known distribution of *Pheidole planifrons*.

#### Taxonomic note.

*Pheidole planifrons* can be identified with the identification key to Northern Vietnamese *Pheidole* provided by Eguchi (2008).

#### Natural history.

*Pheidole planifrons* has been collected from leaf litter in rain forest, limestone forest and rubber plantations. It has also been reported inhabiting in the soil of forest edge and woody habitats (Eguchi 2008).

### Pheidole rugithorax

Taxon classificationAnimaliaHymenopteraFormicidae

Eguchi, 2008

Figure 21

#### Material examined.

CHINA, Yunnan, Xishuangbanna: XTBG (21.919°N, 101.272°E), Secondary forest, 05.vi.2013, 3 workers, 1 Soldier, 550m, Winkler sifting, B. Guénard, B. Blanchard and C. Liu; XTBG (21.911°N, 101.283°E), Limestone forest, 06.vi.2013, 1 worker, 675m, Winkler sifting, B. Guénard, B. Blanchard and C. Liu; XTBG (21.911°N, 101.281°E), Limestone forest, 06.vi.2013, 3 workers, 650m, Winkler sifting, B. Guénard, B. Blanchard and C. Liu; “Holy Hills” (21.920°N, 101.239°E), Rain forest, 07.vi.2013, 2 workers, 665m, Winkler sifting, B. Guénard, B. Blanchard and C. Liu.

#### Distribution.

**Figure 21.**
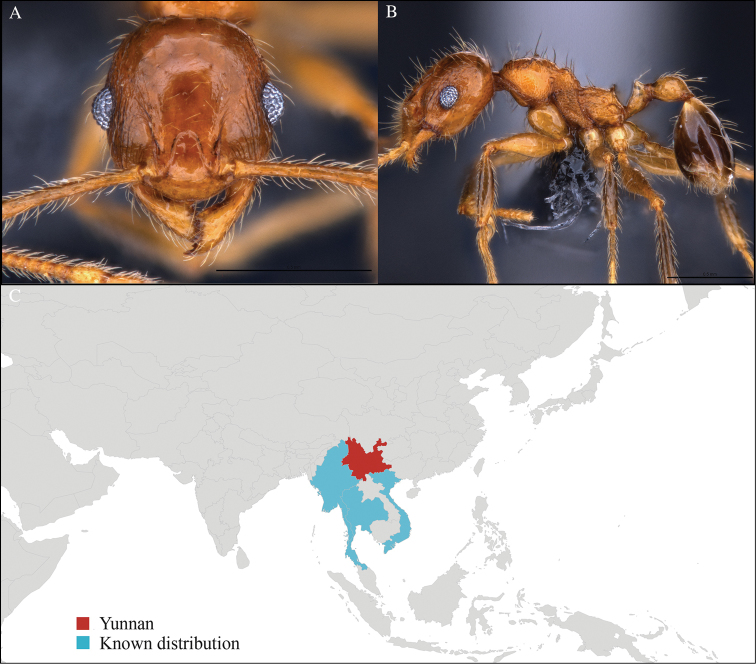
*Pheidole rugithorax* worker, CASENT0717083. **A** Head in front view **B** Mesosoma in profile view **C** Global distribution map.

Yunnan (new record), Vietnam, Myanmar and Thailand (Figure 21C).

#### Taxonomic note.

*Pheidole rugithorax* can be identified with the identification key to Northern Vietnamese *Pheidole* provided by Eguchi (2008).

#### Natural history.

*Pheidole rugithorax* has been collected from leaf litter in rain forest, secondary forest and limestone forest. Otherwise there is no available information on its biology.

### Pheidole smythiesii

Taxon classificationAnimaliaHymenopteraFormicidae

Forel, 1902

Figure 22

#### Material examined.

China, Yunnan, Xishuangbanna: XTBG (21.919°N, 101.272°E), Secondary forest, 05.vi.2013, 9 workers, 550m, Winkler sifting, B. Guénard, B. Blanchard and C. Liu; XTBG (21.918°N, 101.271°E), Secondary forest, 05.vi.2013, 2 workers, 581m, Winkler sifting, B. Guénard, B. Blanchard and C. Liu; XTBG (21.911°N, 101.283°E), Limestone forest, 05.vi.2013, 1 worker, 675m, Winkler sifting, B. Guénard, B. Blanchard and C. Liu; Man Sai village (21.858°N, 101.276°E), Secondary forest, 12.vi.2013, 5 workers, 675m, Winkler sifting, B. Guénard, B. Blanchard and C. Liu.

#### Distribution.

**Figure 22.**
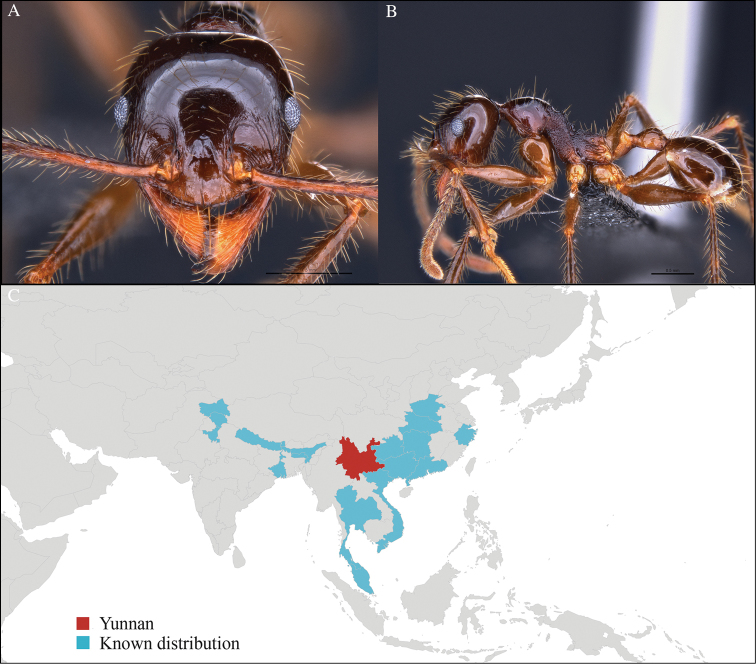
*Pheidole smythiesii* worker, CASENT0713851. **A** Head in front view **B** Mesosoma in profile view **C** Global distribution map.

Widely distributed in South China, Vietnam, Thailand and India (Figure 22C).

#### Taxonomic note.

*Pheidole smythiesii* can be identified with the identification key to Northern Vietnamese *Pheidole* provided by Eguchi (2008).

#### Natural history.

*Pheidole smythiesii* has been collected from leaf litter from secondary forest. Eguchi (2008) reported the species to usually inhabit woody habitats and sometimes open areas where it nests in the soil. *Pheidole smythiesii* is also known to tend aphid colonies (Alfred and Agarwal 1990).

### Pheidole tumida

Taxon classificationAnimaliaHymenopteraFormicidae

Eguchi, 2008

Figure 23

#### Material examined.

CHINA, Yunnan, Xishuangbanna: “Holy Hills” (21.920°N, 101.239°E), Rain forest, 07.vi.2013, 2 workers, 665m, Winkler sifting, B. Guénard, B. Blanchard and C. Liu; Kilometer 55 station (21.966°N, 101.203°E), Rain forest, 13.vi.2013, 2 workers, 825m, Winkler sifting, B. Guénard, B. Blanchard and C. Liu; Kilometer 55 station (21.966°N, 101.203°E), Rain forest, 13.vi.2013, 3 workers, 840m, Winkler sifting, B. Guénard, B. Blanchard and C. Liu; Man Sai village (21.858°N, 101.277°E), Rubber plantation, 12.vi.2013, 4 workers, 705m, Winkler sifting, B. Guénard, B. Blanchard and C. Liu; Man Sai village (21.907°N, 101.273°E), Rubber plantation, 12.vi.2013, 2 workers, 635m, Winkler sifting, B. Guénard, B. Blanchard and C. Liu; Man Sai village (21.857°N, 101.277°E), Rubber plantation, 12.vi.2013, 3 workers, 710m, Winkler sifting, B. Guénard, B. Blanchard and C. Liu; Man Sai village (21.858°N, 101.277°E), Secondary forest, 12.vi.2013, 15 workers, 685m, Winkler sifting, B. Guénard, B. Blanchard and C. Liu; Man Sai village (21.858°N, 101.276°E), Secondary forest, 12.vi.2013, 2 workers, 690m, Winkler sifting, B. Guénard, B. Blanchard and C. Liu; Man Sai village (21.860°N, 101.278°E), Secondary forest, 12.vi.2013, 2 workers, 680m, Winkler sifting, B. Guénard, B. Blanchard and C. Liu; Man Sai village (21.858°N, 101.276°E), Secondary forest, 12.vi.2013, 4 workers, 2 Soldiers, 675m, Winkler sifting, B. Guénard, B. Blanchard and C. Liu; Menglun town (21.934°N, 101.269°E), Rubber plantation, 09.vi.2013, 5 workers, 640m, Winkler sifting, B. Guénard, B. Blanchard and C. Liu; Menglun town (21.933°N, 101.269°E), Rubber plantation, 09.vi.2013, 34 workers, 655m, Winkler sifting, B. Guénard, B. Blanchard and C. Liu; Menglun town (21.932°N, 101.270°E), Rubber plantation, 09.vi.2013, 2 workers, 645m, Winkler sifting, B. Guénard, B. Blanchard and C. Liu; Menglun town (21.932°N, 101.271°E), Rubber plantation, 09.vi.2013, 3 workers, 645m, Winkler sifting, B. Guénard, B. Blanchard and C. Liu; Menglun town (21.931°N, 101.269°E), Rubber plantation, 09.vi.2013, 1 worker, 645m, Winkler sifting, B. Guénard, B. Blanchard and C. Liu; Menglun town (21.933°N, 101.269°E), Rubber plantation, 09.vi.2013, 2 workers, 655m, Winkler sifting, B. Guénard, B. Blanchard and C. Liu; XTBG (21.919°N, 101.274°E), Secondary forest, 05.vi.2013, 1 worker, 552m, Winkler sifting, B. Guénard, B. Blanchard and C. Liu; XTBG (21.919°N, 101.283°E), Secondary forest, 06.vi.2013, 1 worker, 675m, Winkler sifting, B. Guénard, B. Blanchard and C. Liu; XTBG (21.924°N, 101.268°E), Secondary forest, 05.vi.2013, 1 worker, 571m, Winkler sifting, B. Guénard, B. Blanchard and C. Liu; Banna University construction site (21.888°N, 101.266°E), Rubber Plantation, 14.vi.2013, 1 worker, 600m, Winkler sifting, B. Guénard, B. Blanchard and C. Liu; Banna University construction site (21.888°N, 101.266°E), Rubber Plantation, 14.vi.2013, 1 worker, 620m, Winkler sifting, B. Guénard, B. Blanchard and C. Liu; Banna University construction site (21.922°N, 101.268°E), Rubber Plantation, 14.vi.2013, 6 workers, 620m, Winkler sifting, B. Guénard, B. Blanchard and C. Liu.

#### Distribution.

**Figure 23.**
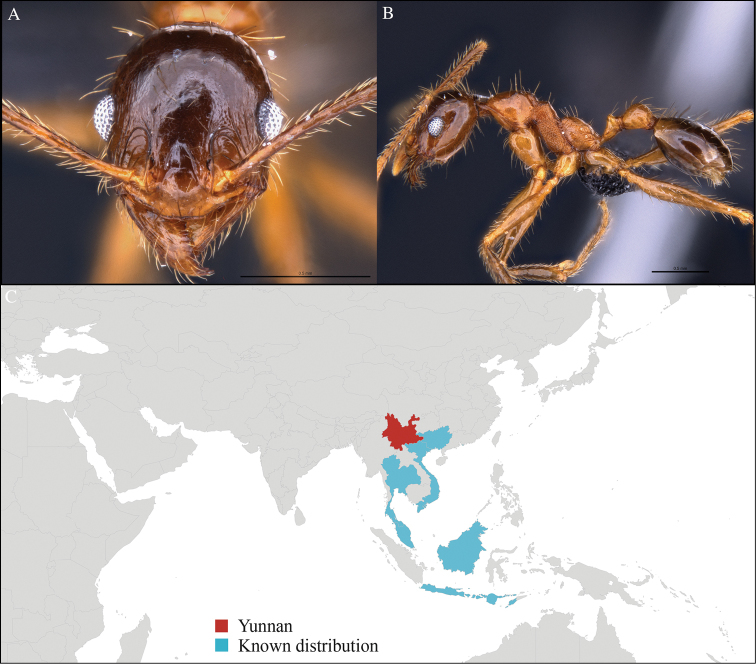
*Pheidole tumida* worker, CASENT0713125. **A** Head in front view **B** Mesosoma in profile view **C** Global distribution map.

Widely distributed in the Australasian and Indo-Malayan subregions (Figure 23C).

#### Taxonomic note.

*Pheidole tumida* can be identified with the identification key to Northern Vietnamese *Pheidole* provided by Eguchi (2008).

#### Natural history.

*Pheidole tumida* has been collected from leaf litter in rain forest, secondary forest and rubber plantation. It has also been reported nesting in the soil and rotting logs of forest edges (Eguchi 2008).

### Pheidole vieti

Taxon classificationAnimaliaHymenopteraFormicidae

Eguchi, 2008

Figure 24

#### Material examined.

CHINA, Yunnan, Xishuangbanna: XTBG (21.912°N, 101.285°E), Limestone forest, 06.vi.2013, 3 workers, 680m, Winkler sifting, B. Guénard, B. Blanchard and C. Liu; XTBG (21.917°N, 101.274°E), Secondary forest, 06.vi.2013, 3 workers, 625m, Winkler sifting, B. Guénard, B. Blanchard and C. Liu; Kilometer 55 station (21.961°N, 101.200°E), Rain forest, 10.vi.2013, 3 workers, 820m, Winkler sifting, B. Guénard, B. Blanchard and C. Liu.

#### Distribution.

**Figure 24.**
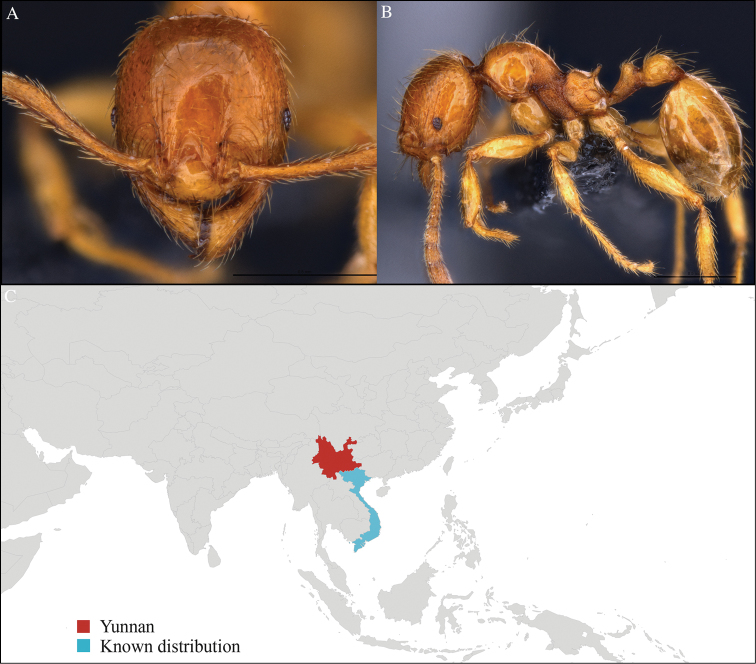
*Pheidole vieti* worker, CASENT0713428. **A** Head in front view **B** Mesosoma in profile view **C** Global distribution map.

Known from Yunnan (new record) and Vietnam (Figure 24C). This new record represents the northern-most occurrence in the distribution of *Pheidole vieti*.

#### Taxonomic note.

*Pheidole vieti* can be identified with the key given provided by Eguchi (2008).

#### Natural history.

*Pheidole vieti* has been collected from leaf litter from rain forest, secondary forest and limestone forest.

### Pheidole zoceana

Taxon classificationAnimaliaHymenopteraFormicidae

Santschi, 1925

Figure 25

#### Material examined.

CHINA, Yunnan, Xishuangbanna: “Holy Hills” (21.920°N, 101.240°E), Secondary forest, 07.vi.2013, 44 workers, 644m, Winkler sifting, B. Guénard, B. Blanchard and C. Liu; “Holy Hills” (21.920°N, 101.239°E), Secondary forest, 07.vi.2013, 5 workers, 665m, Winkler sifting, B. Guénard, B. Blanchard and C. Liu; “Holy Hills” (21.919°N, 101.239°E), Secondary forest, 07.vi.2013, 11 workers, 670m, Winkler sifting, B. Guénard, B. Blanchard and C. Liu; Kilometer 55 station (21.966°N, 101.203°E), Secondary forest, 13.vi.2013, 15 workers, 825m, Winkler sifting, B. Guénard, B. Blanchard and C. Liu; Kilometer 55 station (21.966°N, 101.203°E), Secondary forest, 13.vi.2013, 1 worker, 840m, Winkler sifting, B. Guénard, B. Blanchard and C. Liu; Kilometer 55 station (21.962°N, 101.200°E), Rain forest, 13.vi.2013, 7 workers, 3 Soldiers, 820m, Winkler sifting, B. Guénard, B. Blanchard and C. Liu; Kilometer 55 station (21.960°N, 101.199°E), Rain forest, 13.vi.2013, 25 workers, 840m, Winkler sifting, B. Guénard, B. Blanchard and C. Liu; Kilometer 55 station (21.962°N, 101.200°E), Rain forest, 13.vi.2013, 5 workers, 805m, Winkler sifting, B. Guénard, B. Blanchard and C. Liu; Kilometer 55 station (21.963°N, 101.201°E), Rain forest, 13.vi.2013, 14 workers, 815m, Winkler sifting, B. Guénard, B. Blanchard and C. Liu; Kilometer 55 station (21.964°N, 101.202°E), Rain forest, 13.vi.2013, 2 workers, 820m, Winkler sifting, B. Guénard, B. Blanchard and C. Liu; Man Sai village (21.858°N, 101.276°E), Secondary forest, 12.vi.2013, 2 workers, 690m, Winkler sifting, B. Guénard, B. Blanchard and C. Liu; Man Sai village (21.858°N, 101.276°E), Secondary forest, 12.vi.2013, 1 worker, 675m, Winkler sifting, B. Guénard, B. Blanchard and C. Liu; Menglun town (21.932°N, 101.271°E), Rubber Plantation, 09.vi.2013, 1 worker, 640m, Winkler sifting, B. Guénard, B. Blanchard and C. Liu; XTBG (21.919°N, 101.272°E), Secondary forest, 05.vi.2013, 77 workers, 550m, Winkler sifting, B. Guénard, B. Blanchard and C. Liu; XTBG (21.912°N, 101.285°E), Limestone forest, 05.vi.2013, 22 workers, 680m, Winkler sifting, B. Guénard, B. Blanchard and C. Liu; XTBG (21.919°N, 101.274°E), Secondary forest, 06.vi.2013, 2 workers, 552m, Winkler sifting, B. Guénard, B. Blanchard and C. Liu; XTBG (21.924°N, 101.268°E), Rubber plantation, 05.vi.2013, 3 workers, 571m, Winkler sifting, B. Guénard, B. Blanchard and C. Liu; XTBG (21.918°N, 101.271°E), Secondary forest, 05.vi.2013, 1 worker, 581m, Winkler sifting, B. Guénard, B. Blanchard and C. Liu; XTBG (21.916°N, 101.274°E), Secondary forest, 05.vi.2013, 12 workers, 615m, Winkler sifting, B. Guénard, B. Blanchard and C. Liu.

#### Distribution.

**Figure 25.**
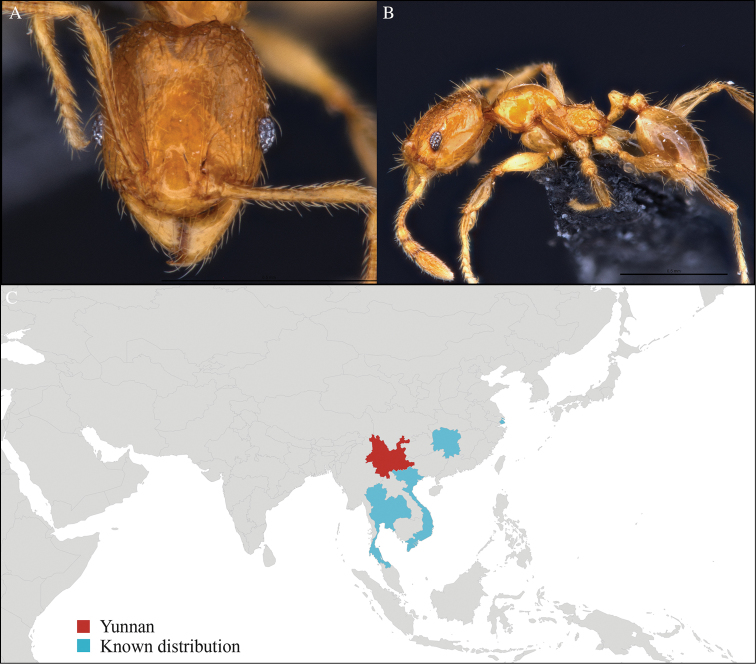
*Pheidole zoceana* worker, CASENT0714742. **A** Head in front view **B** Mesosoma in profile view **C** Global distribution map.

Known from a few localities in China, Vietnam and Thailand (Figure 25C).

#### Taxonomic note.

*Pheidole zoceana* can be identified with the identification key to Northern Vietnamese *Pheidole* provided by Eguchi (2008).

#### Natural history.

*Pheidole zoceana* has been collected from leaf litter in rain forest, secondary forest and rubber plantations. It has also been reported nesting in the soil of forest edges and mountainous area (Eguchi 2008).

### Prenolepis sphingthoraxa

Taxon classificationAnimaliaHymenopteraFormicidae

Zhou & Zheng, 1998

Figure 26

#### Material examined.

CHINA, Yunnan, Xishuangbanna: Kilometer 55 station (21.960°N, 101.199°E), Rain forest, 10.vi.2013, 1 worker, 840m, Winkler sifting, B. Guénard, B. Blanchard and C. Liu.

#### Distribution.

**Figure 26.**
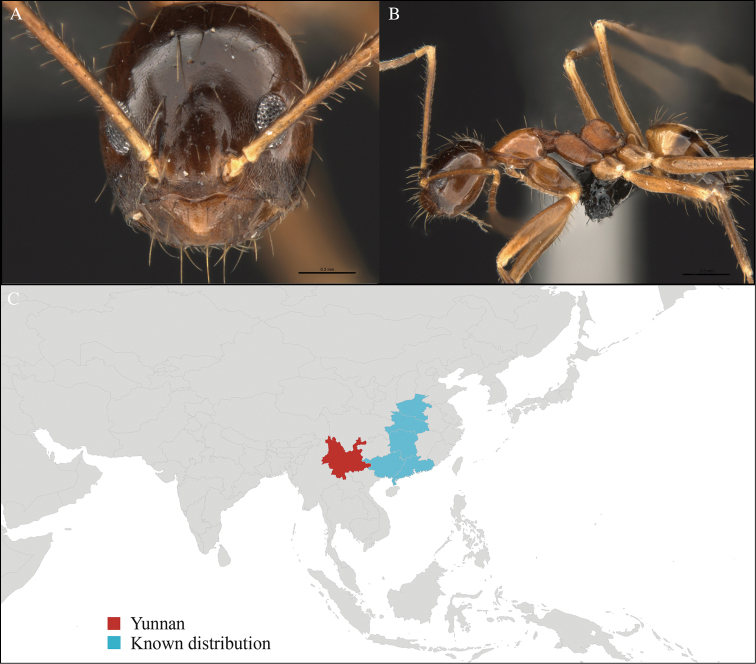
*Prenolepis sphingthoraxa* worker, CASENT0715549. **A** Head in front view **B** Mesosoma in profile view **C** Global distribution map.

Known from Middle and South China (Figure 26C). This new record represents the western-most record in the distribution of *Prenolepis sphingthoraxa*.

#### Taxonomic note.

The identification is based on the original description (Zhou and Zheng 1998).

#### Natural history.

*Prenolepis sphingthoraxa* has been collected from leaf litter in rain forest and little is known about its bionomics.

### Proceratium deelemani

Taxon classificationAnimaliaHymenopteraFormicidae

Perrault, 1981

Figure 27

#### Material examined.

CHINA, Yunnan, Xishuangbanna: Kilometer 55 station (21.964°N, 101.202°E), Rain forest, 13.vi.2013, 1 worker, 820m, Winkler sifting, B. Guénard, B. Blanchard and C. Liu.

#### Distribution.

**Figure 27.**
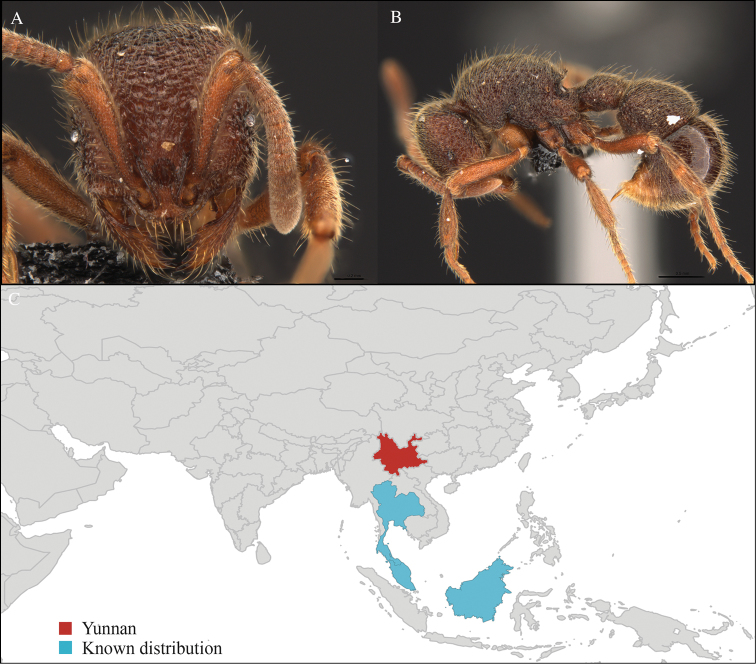
*Proceratium deelemani* worker, CASENT0717686. **A** Head in front view **B** Mesosoma in profile view **C** Global distribution map.

Known from Yunnan (new record), Singapore, Thailand and Borneo (Figure 27C). This new record represents the northern-most record in the distribution of *Proceratium deelemani*.

#### Taxonomic note.

The identification of *Proceratium deelemani* is relatively straightforward with the key provided by Baroni Urbani and De Andrade (2003).

#### Natural history.

*Proceratium deelemani* has been collected from leaf litter in rain forest, and little is known about its bionomics.

### Recurvidris kemneri

Taxon classificationAnimaliaHymenopteraFormicidae

(Wheeler & Wheeler, 1954)

Figure 28

#### Material examined.

CHINA, Yunnan, Xishuangbanna: “Holy Hills” (21.919°N, 101.239°E), Secondary forest, 07.vi.2013, 1 worker, 670m, Winkler sifting, B. Guénard, B. Blanchard and C. Liu; Man Sai village (21.857°N, 101.277°E), Rubber plantation, 12.vi.2013, 3 workers, 710m, Winkler sifting, B. Guénard, B. Blanchard and C. Liu; Man Sai village (21.858°N, 101.276°E), Secondary forest, 12.vi.2013, 1 worker, 685m, Winkler sifting, B. Guénard, B. Blanchard and C. Liu; Man Sai village (21.860°N, 101.278°E), Secondary forest, 12.vi.2013, 1 worker, 680m, Winkler sifting, B. Guénard, B. Blanchard and C. Liu; Kilometer 55 station (21.963°N, 101.201°E), Rain forest, 10.vi.2013, 7 workers, 815m, Winkler sifting, B. Guénard, B. Blanchard and C. Liu.

#### Distribution.

**Figure 28.**
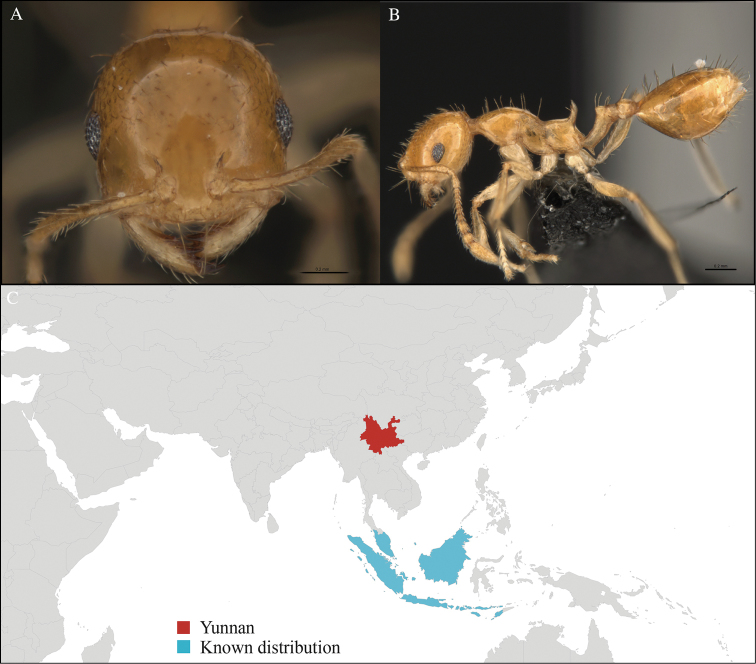
*Recurvidris kemneri* worker, CASENT0715218. **A** Head in front view **B** Mesosoma in profile view **C** Global distribution map.

Widely distributed in the Austral-Asian and Indo-Malayan subregions (Figure 28C). This new northern-most record represents an important extension in the distribution of *Recurvidris kemneri*.

#### Taxonomic note.

The identification is based on Bolton’s (1992) key. Our material from Yunnan fits the re-description in the latter publication very well, even though the propodeal spines seem somewhat longer than in the material from Borneo. However, we consider this as a minor geographic variation.

#### Natural history.

*Recurvidris kemneri* has been collected from leaf litter from rain forest, secondary forest and rubber plantation, and little is known about its bionomics.

### Strumigenys dyschima

Taxon classificationAnimaliaHymenopteraFormicidae

(Bolton, 2000)

Figure 29

#### Material examined.

CHINA, Yunnan, Xishuangbanna: XTBG (21.911°N, 101.283°E), Limestone forest, 06.vi.2013, 2 workers, 675m, Winkler sifting, B. Guénard, B. Blanchard and C. Liu.

#### Distribution.

**Figure 29.**
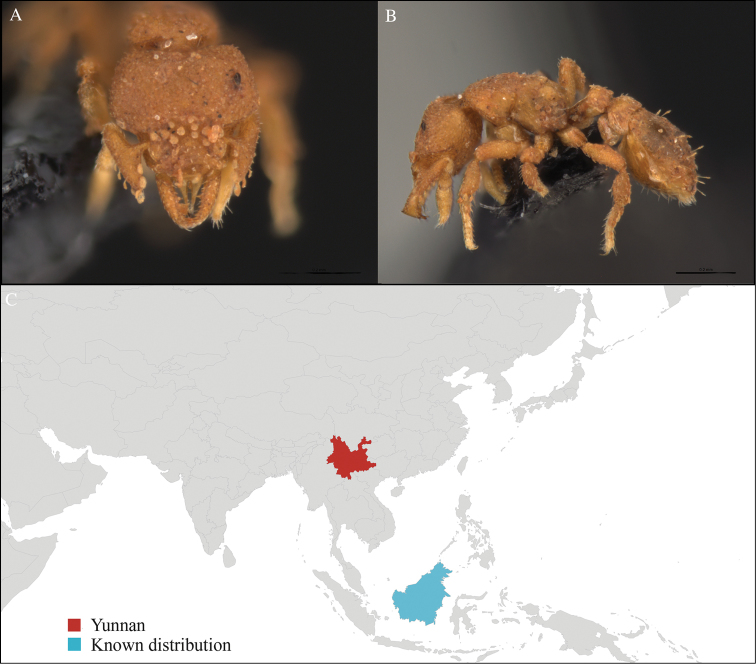
*Strumigenys dyschima* worker, CASENT0717009. **A** Head in front view **B** Mesosoma in profile view **C** Global distribution map.

Known from Yunnan (new record) and Borneo (Figure 29C). This new record represents an important extension in the northern range of the distribution of *Strumigenys dyschima*.

#### Taxonomic note.

*Strumigenys dyschima* can be identified with the identification key given by Bolton (2000; treated as *Pyramica dyschima*).

#### Natural history.

*Strumigenys dyschima* has been collected from leaf litter in limestone forest, and little is known about its bionomics.

### Strumigenys kichijo

Taxon classificationAnimaliaHymenopteraFormicidae

(Terayama, Lin & Wu, 1996)

Figure 30

#### Material examined.

CHINA, Yunnan, Xishuangbanna: XTBG (21.924°N, 101.268°E), Rubber Plantation, 05.vi.2013, 1 worker, 571m, Winkler sifting, B. Guénard, B. Blanchard and C. Liu; Menglun town (21.934°N, 101.269°E), Rubber Plantation, 09.vi.2013, 1 worker, 640m, Winkler sifting, B. Guénard, B. Blanchard and C. Liu.

#### Distribution.

**Figure 30.**
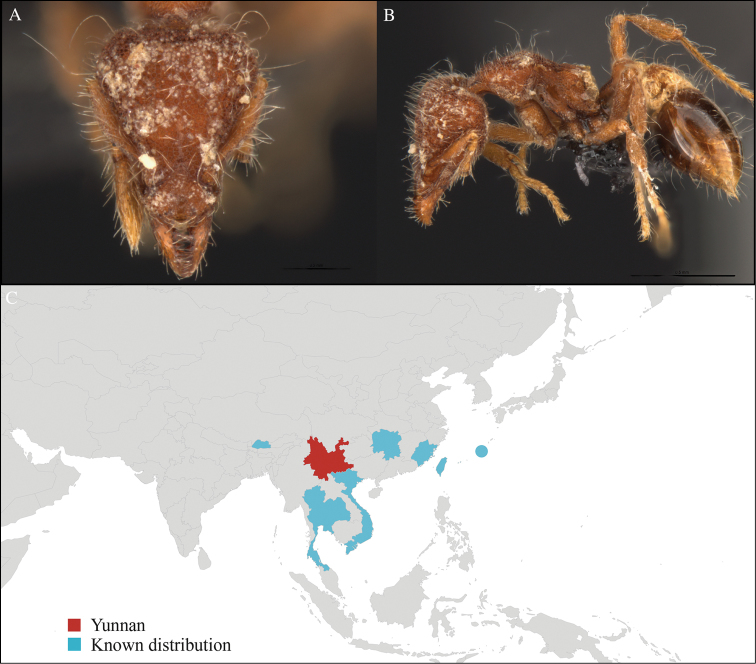
*Strumigenys kichijo* worker, CASENT0713674. **A** Head in front view **B** Mesosoma in profile view **C** Global distribution map.

Widely distributed in Indo-Malayan subregions (Figure 30C).

#### Taxonomic note.

*Strumigenys kichijo* can be identified with the identification key given by Bolton (2000; treated as *Pyramica kichijo*).

#### Natural history.

*Strumigenys kichijo* has been collected from leaf litter in rubber plantations, and little is known about its bionomics.

### Strumigenys mitis

Taxon classificationAnimaliaHymenopteraFormicidae

(Brown, 2000)

Figure 31

#### Material examined.

CHINA, Yunnan, Xishuangbanna: “Holy Hills” (21.920°N, 101.239°E), Secondary forest, 07.vi.2013, 9 workers, 655m, Winkler sifting, B. Guénard, B. Blanchard and C. Liu; “Holy Hills” (21.919°N, 101.239°E), Rain forest, 07.vi.2013, 7 workers, 670m, Winkler sifting, B. Guénard, B. Blanchard and C. Liu; Kilometer 55 station (21.966°N, 101.203°E), Secondary forest, 13.vi.2013, 40 workers, 825m, Winkler sifting, B. Guénard, B. Blanchard and C. Liu; Kilometer 55 station (21.966°N, 101.203°E), Secondary forest, 13.vi.2013, 1 worker, 840m, Winkler sifting, B. Guénard, B. Blanchard and C. Liu; Kilometer 55 station (21.962°N, 101.200°E), Rain forest, 10.vi.2013, 19 workers, 830m, Winkler sifting, B. Guénard, B. Blanchard and C. Liu; Kilometer 55 station (21.961°N, 101.200°E), Rain forest, 10.vi.2013, 8 workers, 820m, Winkler sifting, B. Guénard, B. Blanchard and C. Liu; Kilometer 55 station (21.960°N, 101.199°E), Rain forest, 13.vi.2013, 1 worker, 840m, Winkler sifting, B. Guénard, B. Blanchard and C. Liu; Kilometer 55 station (21.962°N, 101.200°E), Rain forest, 13.vi.2013, 111 worker, 805m, Winkler sifting, B. Guénard, B. Blanchard and C. Liu; Kilometer 55 station (21.963°N, 101.201°E), Rain forest, 13.vi.2013, 122 workers, 815m, Winkler sifting, B. Guénard, B. Blanchard and C. Liu; Kilometer 55 station (21.964°N, 101.202°E), Rain forest, 13.vi.2013, 50 workers, 820m, Winkler sifting, B. Guénard, B. Blanchard and C. Liu; Man Sai village (21.858°N, 101.277°E), Secondary forest, 12.vi.2013, 1 worker, 685m, Winkler sifting, B. Guénard, B. Blanchard and C. Liu; Man Sai village (21.858°N, 101.276°E), Secondary forest, 12.vi.2013, 12 workers, 690m, Winkler sifting, B. Guénard, B. Blanchard and C. Liu; Menglun town (21.932°N, 101.271°E), Rubber plantation, 09.vi.2013, 8 workers, 640m, Winkler sifting, B. Guénard, B. Blanchard and C. Liu; Menglun town (21.932°N, 101.270°E), Rubber plantation, 09.vi.2013, 1 worker, 645m, Winkler sifting, B. Guénard, B. Blanchard and C. Liu; Menglun town (21.931°N, 101.269°E), Rubber plantation, 09.vi.2013, 1 worker, 645m, Winkler sifting, B. Guénard, B. Blanchard and C. Liu; XTBG (21.924°N, 101.268°E), Rubber Plantation, 05.vi.2013, 1 worker, 571m, Winkler sifting, B. Guénard, B. Blanchard and C. Liu; XTBG (21.919°N, 101.272°E), Secondary forest, 05.vi.2013, 82 workers, 550m, Winkler sifting, B. Guénard, B. Blanchard and C. Liu; XTBG (21.919°N, 101.274°E), Secondary forest, 05.vi.2013, 48 workers, 552m, Winkler sifting, B. Guénard, B. Blanchard and C. Liu; XTBG (21.918°N, 101.271°E), Secondary forest, 05.vi.2013, 71 workers, 581m, Winkler sifting, B. Guénard, B. Blanchard and C. Liu; XTBG (21.916°N, 101.274°E), Secondary forest, 08.vi.2013, 2 workers, 615m, Winkler sifting, B. Guénard, B. Blanchard and C. Liu; XTBG (21.917°N, 101.274°E), Secondary forest, 08.vi.2013, 25 workers, 625m, Winkler sifting, B. Guénard, B. Blanchard and C. Liu.

#### Distribution.

**Figure 31.**
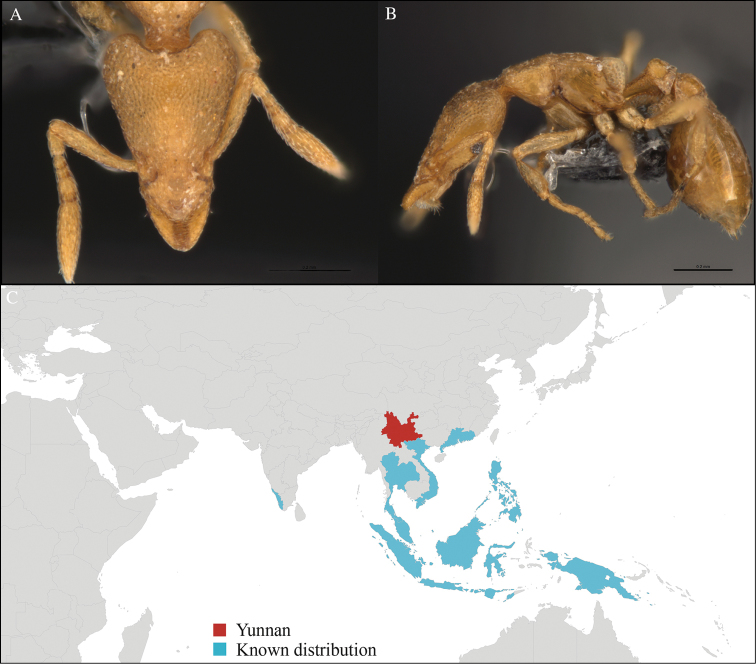
*Strumigenys mitis* worker, CASENT0713676. **A** Head in front view **B** Mesosoma in profile view **C** Global distribution map.

Widely distributed in Austral-Asian and Indo-Malayan subregions (Figure 31C). This new record represents the northern-most known occurrence in the distribution of *Strumigenys mitis*.

#### Taxonomic note.

*Strumigenys mitis* can be identified with the identification key given by Bolton (2000; treated as *Pyramica mitis*) and Bharti (2013, treated as *Pyramica mitis*).

#### Natural history.

*Strumigenys mitis* has been collected from leaf litter in rain forest, secondary forest and rubber plantations, and little is known about its bionomics.

### Strumigenys nepalensis

Taxon classificationAnimaliaHymenopteraFormicidae

Baroni Urbani & De Andrade, 1994

Figure 32

#### Material examined.

CHINA, Yunnan, Xishuangbanna: “Holy Hills” (21.920°N, 101.240°E), Secondary forest, 07.vi.2013, 5 workers, 655m, Winkler sifting, B. Guénard, B. Blanchard and C. Liu; “Holy Hills” (21.920°N, 101.239°E), Secondary forest, 07.vi.2013, 12 workers, 665m, Winkler sifting, B. Guénard, B. Blanchard and C. Liu; “Holy Hills” (21.919°N, 101.239°E), Rain forest, 07.vi.2013, 1 worker, 670m, Winkler sifting, B. Guénard, B. Blanchard and C. Liu; Kilometer 55 station (21.963°N, 101.201°E), Rain forest, 13.vi.2013, 2 workers, 815m, Winkler sifting, B. Guénard, B. Blanchard and C. Liu; Man Sai village (21.858°N, 101.277°E), Rubber Plantation, 12.vi.2013, 2 workers, 705m, Winkler sifting, B. Guénard, B. Blanchard and C. Liu; Man Sai village (21.857°N, 101.277°E), Rubber Plantation, 12.vi.2013, 2 workers, 710m, Winkler sifting, B. Guénard, B. Blanchard and C. Liu; Man Sai village (21.858°N, 101.277°E), Secondary forest, 12.vi.2013, 2 workers, 685m, Winkler sifting, B. Guénard, B. Blanchard and C. Liu; Man Sai village (21.858°N, 101.276°E), Secondary forest, 12.vi.2013, 3 workers, 690m, Winkler sifting, B. Guénard, B. Blanchard and C. Liu; Man Sai village (21.858°N, 101.276°E), Secondary forest, 12.vi.2013, 4 workers, 685m, Winkler sifting, B. Guénard, B. Blanchard and C. Liu; Man Sai village (21.860°N, 101.278°E), Secondary forest, 12.vi.2013, 6 workers, 680m, Winkler sifting, B. Guénard, B. Blanchard and C. Liu; Man Sai village (21.858°N, 101.276°E), Secondary forest, 12.vi.2013, 1 worker, 675m, Winkler sifting, B. Guénard, B. Blanchard and C. Liu; XTBG (21.919°N, 101.272°E), Secondary forest, 05.vi.2013, 57 workers, 550m, Winkler sifting, B. Guénard, B. Blanchard and C. Liu; XTBG (21.912°N, 101.285°E), Limestone forest, 06.vi.2013, 1 worker, 680m, Winkler sifting, B. Guénard, B. Blanchard and C. Liu; XTBG (21.919°N, 101.274°E), Secondary forest, 05.vi.2013, 13 workers, 552m, Winkler sifting, B. Guénard, B. Blanchard and C. Liu; XTBG (21.911°N, 101.283°E), Limestone forest, 06.vi.2013, 7 workers, 675m, Winkler sifting, B. Guénard, B. Blanchard and C. Liu; XTBG (21.912°N, 101.282°E), Limestone forest, 06.vi.2013, 21 workers, 640m, Winkler sifting, B. Guénard, B. Blanchard and C. Liu; XTBG (21.911°N, 101.281°E), Limestone forest, 06.vi.2013, 21 workers, 650m, Winkler sifting, B. Guénard, B. Blanchard and C. Liu; Banna University construction site (21.888°N, 101.266°E), Rubber plantation, 14.vi.2013, 3 workers, 600m, Winkler sifting, B. Guénard, B. Blanchard and C. Liu; Banna University construction site (21.888°N, 101.266°E), Rubber plantation, 14.vi.2013, 6 workers, 620m, Winkler sifting, B. Guénard, B. Blanchard and C. Liu; Banna University construction site (21.922°N, 101.268°E), Rubber plantation, 14.vi.2013, 1 worker, 620m, Winkler sifting, B. Guénard, B. Blanchard and C. Liu.

#### Distribution.

**Figure 32.**
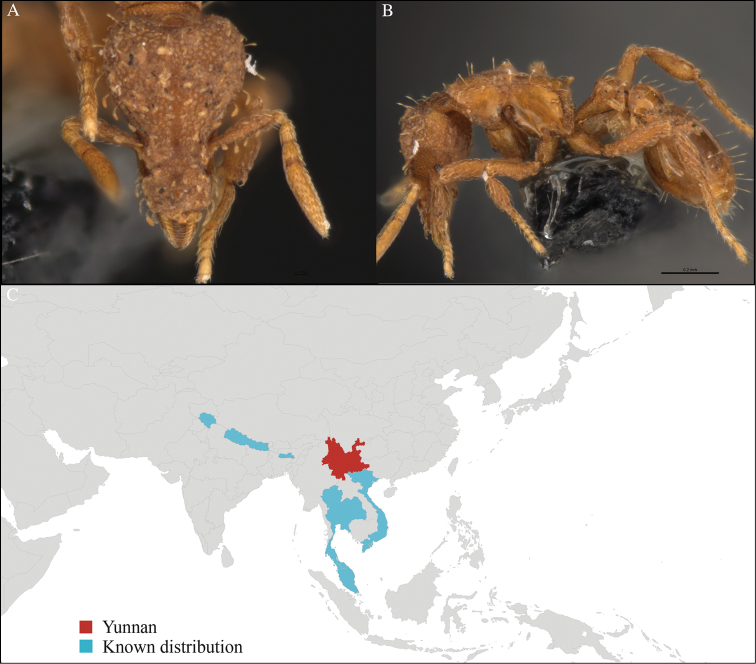
*Strumigenys nepalensis* worker, CASENT0715046. **A** Head in front view **B** Mesosoma in profile view **C** Global distribution map.

Known from Yunnan (new record), North Indian, Vietnam and Thailand (Figure 32C).

#### Taxonomic note.

*Strumigenys nepalensis* can be identified with the identification key given by Bolton (2000; treated as *Pyramica nepalensis*).

#### Natural history.

*Strumigenys nepalensis* has been collected from leaf litter in rain forest, secondary forest, limestone forest and rubber plantations, and little is known about its bionomics.

### Strumigenys rallarhina

Taxon classificationAnimaliaHymenopteraFormicidae

Bolton, 2000

Figure 33

#### Material examined.

CHINA, Yunnan, Xishuangbanna: XTBG (21.919°N, 101.272°E), Secondary forest, 05.vi.2013, 121 workers, 550m, Winkler sifting, B. Guénard, B. Blanchard and C. Liu; XTBG (21.919°N, 101.274°E), Secondary forest, 05.vi.2013, 34 workers, 552m, Winkler sifting, B. Guénard, B. Blanchard and C. Liu; XTBG (21.918°N, 101.271°E), Secondary forest, 05.vi.2013, 35 workers, 581m, Winkler sifting, B. Guénard, B. Blanchard and C. Liu; XTBG (21.916°N, 101.274°E), Secondary forest, 08.vi.2013, 7 workers, 615m, Winkler sifting, B. Guénard, B. Blanchard and C. Liu; XTBG (21.917°N, 101.274°E), Secondary forest, 08.vi.2013, 44 workers, 625m, Winkler sifting, B. Guénard, B. Blanchard and C. Liu; Kilometer 55 station (21.962°N, 101.200°E), Rain forest, 10.vi.2013, 22 workers, 830m, Winkler sifting, B. Guénard, B. Blanchard and C. Liu; Kilometer 55 station (21.961°N, 101.200°E), Rain forest, 10.vi.2013, 15 workers, 820m, Winkler sifting, B. Guénard, B. Blanchard and C. Liu; Kilometer 55 station (21.960°N, 101.199°E), Rain forest, 10.vi.2013, 26 workers, 840m, Winkler sifting, B. Guénard, B. Blanchard and C. Liu; Kilometer 55 station (21.962°N, 101.200°E), Rain forest, 13.vi.2013, 9 workers, 805m, Winkler sifting, B. Guénard, B. Blanchard and C. Liu; Kilometer 55 station (21.964°N, 101.202°E), Rain forest, 13.vi.2013, 16 workers, 820m, Winkler sifting, B. Guénard, B. Blanchard and C. Liu.

#### Distribution.

**Figure 33.**
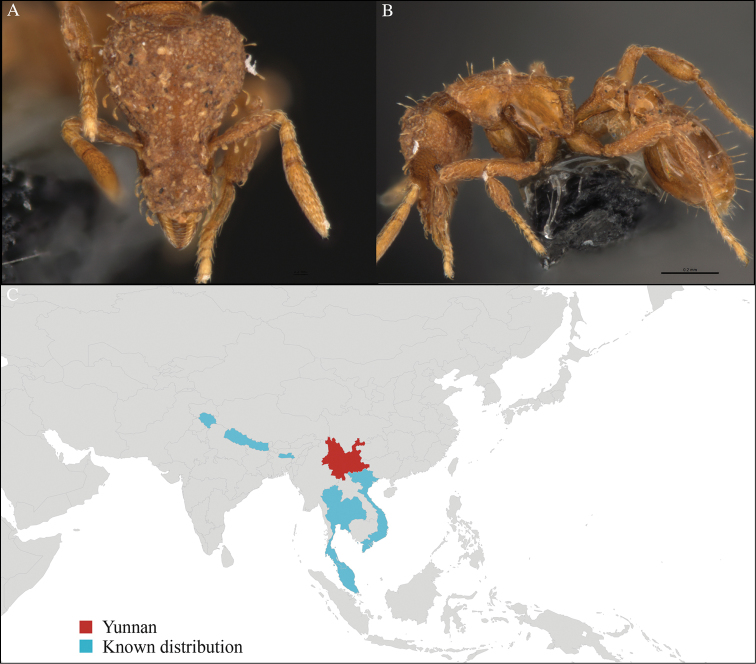
*Strumigenys rallarhina* worker, CASENT0715395. **A** Head in front view **B** Mesosoma in profile view **C** Global distribution map.

Known from Yunnan (new record), Guangxi and Vietnam (Figure 33C).

#### Taxonomic note.

*Strumigenys rallarhina* can be identified with the identification key provided by Bolton (2000).

#### Natural history.

*Strumigenys rallarhina* has been collected from leaf litter in rain forest and secondary forest, and little is known about its bionomics.

### Strumigenys sauteri

Taxon classificationAnimaliaHymenopteraFormicidae

(Forel, 1912)

Figure 34

#### Material examined.

CHINA, Yunnan, Xishuangbanna: XTBG (21.918°N, 101.271°E), Secondary forest, 05.vi.2013, 10 workers, 581m, Winkler sifting, B. Guénard, B. Blanchard and C. Liu; XTBG (21.917°N, 101.274°E), Secondary forest, 08.vi.2013, 3 workers, 625m, Winkler sifting, B. Guénard, B. Blanchard and C. Liu; Kilometer 55 station (21.962°N, 101.200°E), Rain forest, 13.vi.2013, 9 workers, 805m, Winkler sifting, B. Guénard, B. Blanchard and C. Liu; Kilometer 55 station (21.963°N, 101.201°E), Rain forest, 13.vi.2013, 3 workers, 815m, Winkler sifting, B. Guénard, B. Blanchard and C. Liu.

#### Distribution.

**Figure 34.**
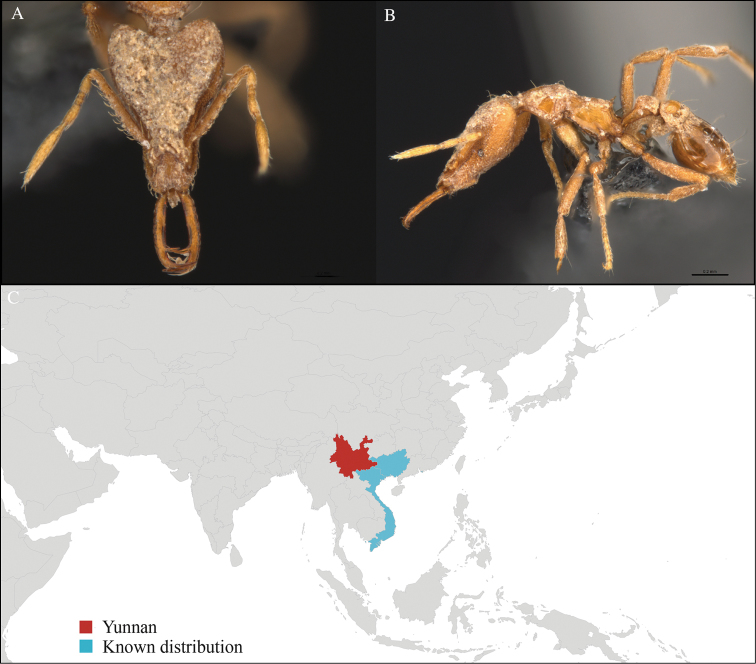
*Strumigenys sauteri* worker, CASENT0717023. **A** Head in front view **B** Mesosoma in profile view **C** Global distribution map.

Widely distributed in Indo-Malayan subregions (Figure 34C).

#### Taxonomic note.

*Strumigenys sauteri* can be identified with the identification key given by Bolton (2000; treated as *Pyramica sauteri*).

#### Natural history.

*Strumigenys sauteri* has been collected from leaf litter in rain forest and secondary forest, and little is known about its bionomics.

### Technomyrmex pratensis

Taxon classificationAnimaliaHymenopteraFormicidae

(Smith, 1860)

Figure 35

#### Material examined.

CHINA, Yunnan, Xishuangbanna: XTBG (21.918°N, 101.271°E), Secondary forest, 05.vi.2013, 4 workers, 581 m, Winkler sifting, B. Guénard, B. Blanchard and C. Liu; XTBG (21.919°N, 101.274°E), Secondary forest, 11.vi.2013, 4 workers, 590 m, Hand collection, B. Guénard, B. Blanchard and C. Liu.

#### Distribution.

**Figure 35.**
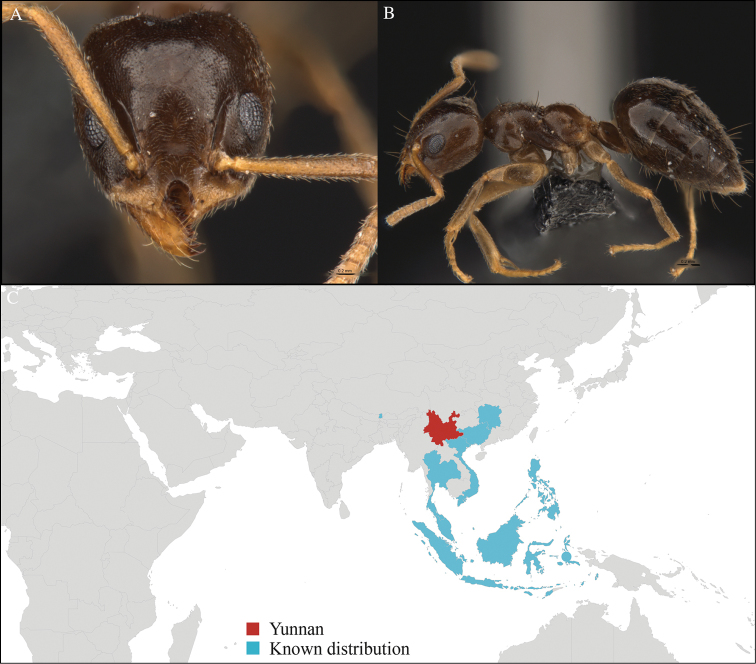
*Technomyrmex pratensis* worker, CASENT0715863. **A** Head in front view **B** Mesosoma in profile view **C** Global distribution map.

Widely distributed in the Austral-Asian and Indo-Malayan subregions (Figure 35C).

#### Taxonomic note.

*Technomyrmex pratensis* is the only member of the *Technomyrmex pratensis* species group. It is a very conspicuous species within the genus, and its identification is very easy with the key provided by Bolton (2007).

#### Natural history.

*Technomyrmex pratensis* has been collected from leaf litter in secondary forest, and little is known about its bionomics.

### Tetramorium difficile

Taxon classificationAnimaliaHymenopteraFormicidae

Bolton, 1977

Figure 36

#### Material examined.

CHINA, Yunnan, Xishuangbanna: XTBG (21.918°N, 101.271°E), Secondary forest, 05.vi.2013, 2 workers, 552 m, Winkler sifting, B. Guénard, B. Blanchard and C. Liu.

#### Distribution.

**Figure 36.**
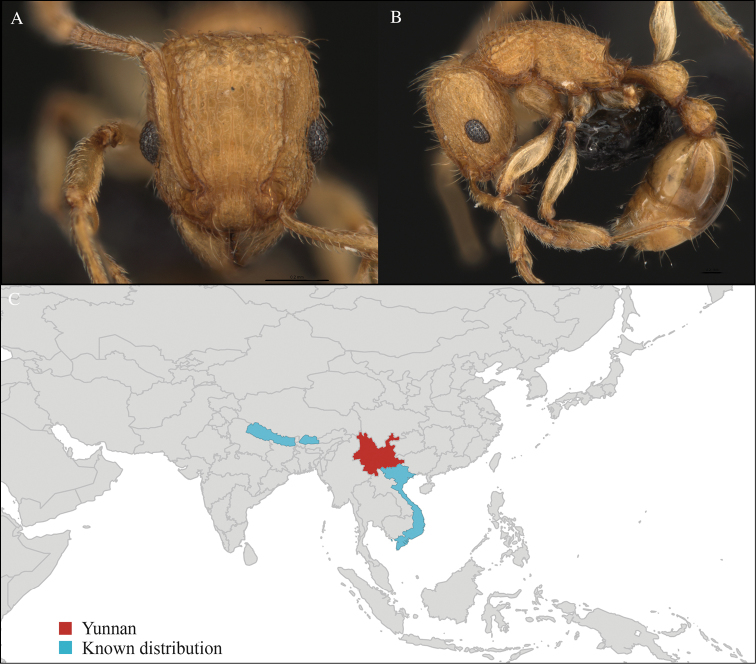
*Tetramorium difficile* worker, CASENT0713193. **A** Head in front view **B** Mesosoma in profile view **C** Global distribution map.

Known form Yunnan (new record), northern India, and Vietnam (Figure 36C).

#### Taxonomic note.

*Tetramorium difficile* is a member of the *Tetramorium tonganum* group and can be identified with the key provided by Bolton (1977). However, *Tetramorium difficile* under its current definition is morphologically very close to *Tetramorium tonganum*. It is likely that both are conspecific and the material listed as *Tetramorium difficile* represents intraspecific forms of the very widespread *Tetramorium tonganum*.

#### Natural history.

*Tetramorium difficile* has been collected from leaf litter in secondary forest, and little is known about its bionomics.

### Tetramorium flavipes

Taxon classificationAnimaliaHymenopteraFormicidae

Emery, 1893

Figure 37

#### Material examined.

CHINA, Yunnan, Xishuangbanna: XTBG (21.918°N, 101.271°E), Secondary forest, 05.vi.2013, 35 workers, 552 m, Winkler sifting, B. Guénard, B. Blanchard and C. Liu; XTBG (21.917°N, 101.274°E), Secondary forest, 08.vi.2013, 33 workers, 625 m, Winkler sifting, B. Guénard, B. Blanchard and C. Liu; Kilometer 55 station (21.961°N, 101.201°E), Rain forest, 10.vi.2013, 8 workers, 820m, Winkler sifting, B. Guénard, B. Blanchard and C. Liu; Kilometer 55 station (21.963°N, 101.200°E), Rain forest, 13.vi.2013, 5 workers, 815m, Winkler sifting, B. Guénard, B. Blanchard and C. Liu.

#### Distribution.

**Figure 37.**
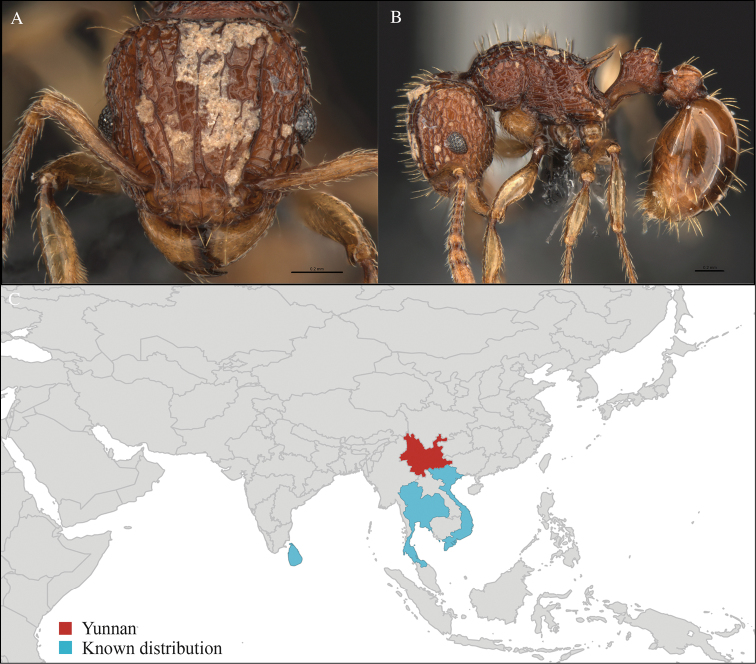
*Tetramorium flavipes* worker, CASENT0713761. **A** Head in front view **B** Mesosoma in profile view **C** Global distribution map.

Known from Yunnan (new record), Vietnam, Thailand and Sri Lanka (Figure 37C). This new record represents the northern-most record in the distribution of this species.

#### Taxonomic note.

*Tetramorium flavipes* is a member of the *Tetramorium tortuosum* group. Its identification is relatively straightforward with the key given by Bolton (1977). However, *Tetramorium flavipes*, originally described from Thailand, is very close to *Tetramorium eleates* Forel, 1913 from Borneo and the Philippines, and as already pointed out by Bolton (1977), both could represent geographic variants of the same species.

#### Natural history.

*Tetramorium flavipes* has been collected from leaf litter in secondary forest, and very little is known about its bionomics.

### Tetramorium parvispinum

Taxon classificationAnimaliaHymenopteraFormicidae

(Emery, 1893)

Figure 38

#### Material examined.

CHINA, Yunnan, Xishuangbanna: XTBG (21.919°N, 101.274°E), Secondary forest, 05.vi.2013, 155 workers, 550 m, Winkler sifting, B. Guénard, B. Blanchard and C. Liu; XTBG (21.924°N, 101.268°E), Rubber plantation, 05.vi.2013, 6 workers, 571 m, Winkler sifting, B. Guénard, B. Blanchard and C. Liu; XTBG (21.917°N, 101.270°E), Secondary forest, 05.vi.2013, 7 workers, 580 m, Winkler sifting, B. Guénard, B. Blanchard and C. Liu; XTBG (21.911°N, 101.281°E), Limestone rain forest, 06.vi.2013, 155 workers, 650 m, Winkler sifting, B. Guénard, B. Blanchard and C. Liu; XTBG (21.916°N, 101.274°E), Secondary forest, 08.vi.2013, 58 workers, 615 m, Winkler sifting, B. Guénard, B. Blanchard and C. Liu; Menglun town (21.930°N, 101.269°E), Rubber plantation, 09.vi.2013, 2 workers, 640 m, Winkler sifting, B. Guénard, B. Blanchard and C. Liu; XTBG (21.890°N, 101.267°E), Rubber plantation, 14.vi.2013, 9 workers, 620 m, Winkler sifting, B. Guénard, B. Blanchard and C. Liu.

#### Distribution.

**Figure 38.**
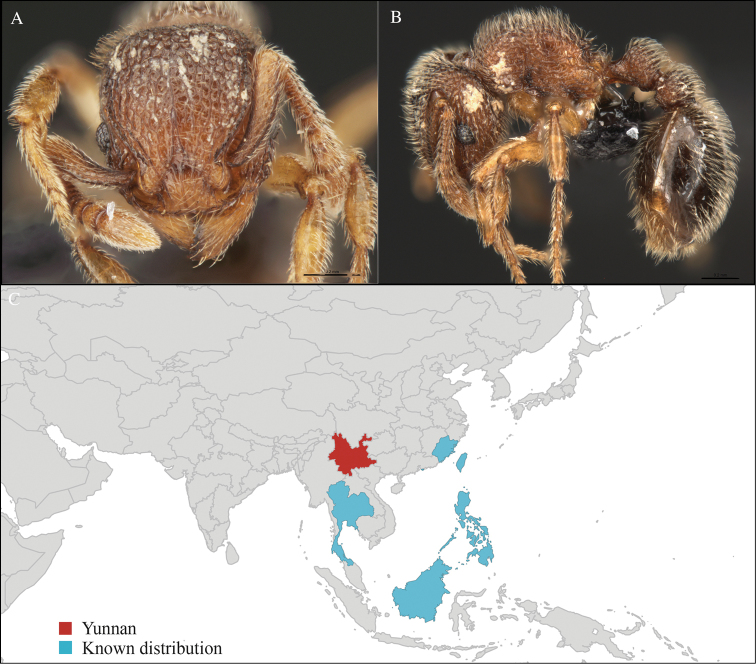
*Tetramorium parvispinum* worker, CASENT0735806. **A** Head in front view **B** Mesosoma in profile view **C** Global distribution map.

Widely distributed in the Indo-Malayan subregion (Figure 38C).

#### Taxonomic note.

*Tetramorium parvispinum* is a member of the *Tetramorium walshi* species group. It can be identified with the key presented by Bolton (1976; as *Triglyphothrix parvispina*)

#### Natural history.

*Tetramorium parvispinum* has been collected from leaf litter in secondary forest, limestone forest and rubber plantations, and little is known about its bionomics.

### Tetramorium polymorphum

Taxon classificationAnimaliaHymenopteraFormicidae

Yamane & Jaitrong, 2011

Figure 39

#### Material examined.

CHINA, Yunnan, Xishuangbanna: XTBG (21.917°N, 101.274°E), Rain forest, 05.vi.2013, 1 major worker, 552 m, Winkler sifting, B. Guénard, B. Blanchard and C. Liu; XTBG (21.918°N, 101.270°E), Rain forest, 05.vi.2013, 3 workers, 581 m, Winkler sifting, B. Guénard, B. Blanchard and C. Liu; XTBG (21.919°N, 101.272°E), Rain forest, 05.vi.2013, 10 workers, 550 m, Winkler sifting, B. Guénard, B. Blanchard and C. Liu; “Holy Hills” (21.920°N, 101.239°E), Rain forest, 07.vi.2013, 10 worker, 665m, Winkler sifting, B. Guénard, B. Blanchard and C. Liu; XTBG (21.928°N, 101.256°E), Rain forest, 07.vi.2013, 10 workers, 565 m, Hand collection, B. Guénard, B. Blanchard and C. Liu; Man Sai village (21.860°N, 101.278°E), Rain forest, 12.vi.2013, 1 worker, 680 m, Winkler sifting, B. Guénard, B. Blanchard and C. Liu.

#### Distribution.

**Figure 39.**
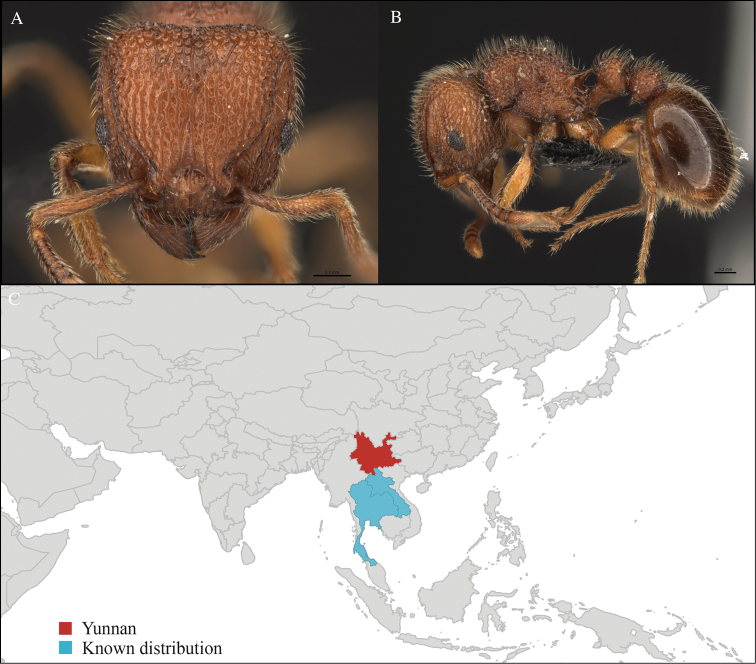
*Tetramorium polymorphum* worker, CASENT0713055. **A** Head in front view **B** Mesosoma in profile view **C** Global distribution map.

Known from Yunnan (new record), Laos and Thailand (Figure 39C). This new record represents the northern-most record in the distribution of *Tetramorium polymorphum*.

#### Taxonomic note.

*Tetramorium polymorphum* is a member of the *Tetramorium walshi* species group. Its identification is not easy since the species was not known when Bolton (1976) published his revision of the genus *Triglyphothrix* (now Tetramorium), in which he provided keys to the Indo-Malayan and Austral-Asian *Tetramorium walshi* and *Tetramorium obesum* species groups. However, by combining Bolton’s (1976) work with the recent species description of Yamane and Jaitrong (2011) the identification is relatively straightforward. It is very similar to the closely related and sympatric *Tetramorium kheperra* Bolton, 1976, and the identification key of Bolton (1976) will lead the user to that species. The recent addition to Bolton’s key provided by Yamane and Jaitrong (2011) clearly separates both species.

#### Natural history.

*Tetramorium polymorphum* is a very special member of the genus *Tetramorium* since it is the only known species that possesses a polymorphic worker caste divisible into distinctive minor, media and major workers (Yamane and Jaitrong 2011). Yamane and Jaitrong (2011) also report that this species is comparatively aggressive and hypothesize that the major worker could have a defensive function. In addition, they emphasize that *Tetramorium polymorphum* is only found in undisturbed rain forest habitats in Thailand and Laos. Our data from Yunnan supports this since it was predominantly sampled from rain forest.

### Tetramorium tonganum

Taxon classificationAnimaliaHymenopteraFormicidae

Mayr, 1870

Figure 40

#### Material examined.

CHINA, Yunnan, Xishuangbanna: XTBG (21.919°N, 101.274°E), Secondary forest, 05.vi.2013, 9 workers, 552 m, Winkler sifting, B. Guénard, B. Blanchard and C. Liu; Menglun town (21.934°N, 101.269°E), Secondary forest, 09.vi.2013, 2 workers, 640 m, Winkler sifting, B. Guénard, B. Blanchard and C. Liu.

#### Distribution.

**Figure 40.**
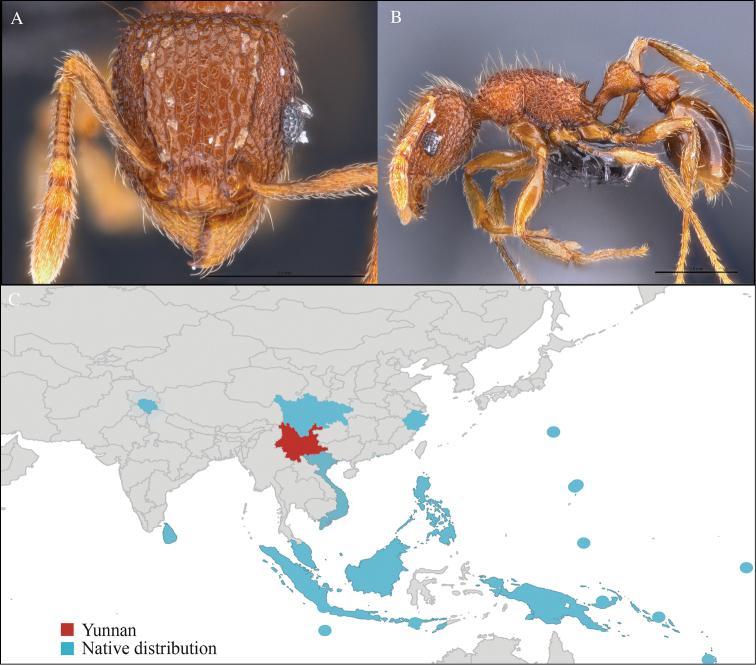
*Tetramorium tonganum* worker, CASENT0713454. **A** Head in front view **B** Mesosoma in profile view **C** Global distribution map.

*Tetramorium tonganum* is widely distributed in the Austral-Asian and Indo-Malayan subregions where it ranges from western Oceania to South East Asia (Figure 40C). Bolton (1977) has noted already that the species is widespread in its native range and has the characteristics of tramp species. It is very likely that future collections will reveal its presence in more Chinese provinces Southeast Asian countries.

#### Taxonomic note.

*Tetramorium polymorphum* belongs to the *Tetramorium tonganum* group and can be easily identified with the key provided by Bolton (1977).

#### Natural history.

*Tetramorium polymorphum* has been collected from leaf litter in secondary forest, and is known to be an exotic species in China (Guenard and Dunn 2012). Despite its wide distribution and tramping ability, there is very little information about its biology. In addition to Bolton (1977), Sarnat and Economo (2012) also confirm that *Tetramorium tonganum* is able to establish populations outside its native range without damaging or significantly altering ecological or agricultural systems in its introduced habitats. They also report that *Tetramorium tonganum* is mostly found on vegetation in disturbed or edge habitats.

## Discussion

The total number of named ant species in China is 939, but the true species richness is expected to be significantly higher, perhaps as high as 1200 to 1600 species (Guénard and Dunn 2012). The collection of these 40 new ant records for Yunnan and 17 for China through Winkler extraction, combined with the discovery of the extremely rare ant species *Bannapone scrobiceps* (Guenard et al. 2013), should encourage myrmecologists to consider leaf litter extraction as one of the primary methods to collect leaf litter ants, especially for places where this method has not previously been used. Nevertheless, further sampling methods that specifically target different strata will very likely yield additional species, which is especially true for hypogaeic and arboreal ant communities.

Based on our collections, many newly recorded species, such as *Discothyrea clavicornis*, *Myrmecina curvispina*, and *Odontoponera denticulata* are relatively common. The reason why those species were never reported from Yunnan before may be due to different collection techniques and/or misidentifications. For example, *Odontoponera denticulata* has long been misidentified as *Odontoponera transversa* (Yamane 2009). Another reason may be that some of the newly recorded species have been described only recently outside of Yunnan and/or China, such as *Myrmecina curvispina* and *Pheidole tumida* (Zhou et al. 2008, Eguchi 2008).

Many new species records in our collection such as *Aenictus artipus*, *Aenictus maneerati*, *Aenictus paradentatus*, *Discothyrea clavicornis*, *Dolichoderus laotius*, *Gesomyrmex kalshoveni*, *Gnamptogenys treta*, *Pheidole plagiaria*, *Pheidole planifrons*, *Pheidole rugithorax*, *Pheidole tumida*, *Pheidole vieti*, *Recurvidris kemneri*, *Strumigenys dyschima*, *Strumigenys mitis*, *Tetramorium difficile*, *Tetramorium flavipes*, *Tetramorium parvispinum*, and *Tetramorium tonganum*, are at the northern limit of their known distribution in Yunnan. Interestingly, the occurrence of several species in Yunnan, such as *Discothyrea clavicornis*, *Gesomyrmex kalshoveni*, *Gnamptogenys treta*, *Recurvidris kemneri*, and *Strumigenys dyschima* constitutes a disjunction from the rest of their known distribution in the Malay Peninsula. At present, it is unclear if these represent sampling artifacts and the ranges are actually continuous in the region, if these species ranges represent true biogeographic disjunctions, or if they are actually different species. Only future diversity inventories and taxonomic treatments, of which this paper represents one modest contribution, can answer these questions and further resolve the biodiversity map for ants and other organisms.

Despite the comparatively short collecting time we invested in the inventory of the myrmecofauna, we were able to identify 145 species, of which over 30% represent new records. This increases the list of known species for Yunnan by 10%, and there are still more than 60 species that we tentatively consider undescribed. This shows how little was previously known about the ant fauna of the region, and we are convinced that more intensive sampling in different habitats and microhabitats will likely reveal the presence of even more species or help improve the current taxonomic resolution. In this context, we think that Yunnan should be considered an area of high biodiversity value and deserving of attention of both biologists and conservationists. Regrettably, this interesting biota is being degraded at an alarming speed, particularly due to the rapid expansion of rubber plantations in the area (Li et al. 2007).

## Supplementary Material

XML Treatment for Aenictus artipus

XML Treatment for Aenictus hodgsoni

XML Treatment for Aenictus maneerati

XML Treatment for Aenictus paradentatus

XML Treatment for Aenictus thailandianus

XML Treatment for Bannapone scrobiceps

XML Treatment for Carebara melasolena

XML Treatment for Discothyrea clavicornis

XML Treatment for Discothyrea kamiteta

XML Treatment for Dolichoderus laotius

XML Treatment for Echinopla cherapunjiensis

XML Treatment for Gesomyrmex kalshoveni

XML Treatment for Gnamptogenys costata

XML Treatment for Gnamptogenys treta

XML Treatment for Myrmecina curvispina

XML Treatment for Myrmecina guangxiensis

XML Treatment for Odontoponera denticulata

XML Treatment for Pheidole hongkongensis

XML Treatment for Pheidole plagiaria

XML Treatment for Pheidole planifrons

XML Treatment for Pheidole rugithorax

XML Treatment for Pheidole smythiesii

XML Treatment for Pheidole tumida

XML Treatment for Pheidole vieti

XML Treatment for Pheidole zoceana

XML Treatment for Prenolepis sphingthoraxa

XML Treatment for Proceratium deelemani

XML Treatment for Recurvidris kemneri

XML Treatment for Strumigenys dyschima

XML Treatment for Strumigenys kichijo

XML Treatment for Strumigenys mitis

XML Treatment for Strumigenys nepalensis

XML Treatment for Strumigenys rallarhina

XML Treatment for Strumigenys sauteri

XML Treatment for Technomyrmex pratensis

XML Treatment for Tetramorium difficile

XML Treatment for Tetramorium flavipes

XML Treatment for Tetramorium parvispinum

XML Treatment for Tetramorium polymorphum

XML Treatment for Tetramorium tonganum
